# Global incidence pattern and factors associated with common cutaneous reactions related to COVID-19 vaccination of 2.55 million participants in real-world settings: A systematic review and meta-analysis

**DOI:** 10.7189/jogh.13.06008

**Published:** 2023-02-10

**Authors:** Yang Guo, Xue-Shan Cao, Hua-Tong Yang, Meng-Ge Zhou, Bo Yu

**Affiliations:** 1 Department of Dermatology, Institute of Dermatology, Peking University Shenzhen Hospital, Shenzhen Peking University-The Hong Kong University of Science and Technology Medical Center, Shenzhen, China; 2 College of Life Science and Oceanography, Shenzhen University, Shenzhen, China; 3 Department of Statistics and Data Science, Southern University of Science and Technology, Shenzhen, China; 4 Department of Statistics, University of California Berkeley, Berkeley, California, USA; 5 Department of Epidemiology and Biostatistics, Institute of Basic Medical Sciences Chinese Academy of Medical Sciences, School of Basic Medicine, Peking Union Medical College, Beijing, China

## Abstract

**Background:**

Understanding the incidence pattern of cutaneous reactions is crucial for promoting COVID-19 vaccination. We aimed to report the global incidence pattern of, and factors associated with common cutaneous reactions related to COVID-19 vaccination in real-world settings.

**Methods:**

We searched five databases (PubMed, Web of Science, Embase, CNKI, and Wanfang) from inception to May 13, 2022, for studies reporting the incidence of common cutaneous reactions related to COVID-19 vaccines in real-world settings. The outcomes were the systematic skin reactions (rash and urticaria) and the local injection site reactions (pain, swelling, redness, and erythema). We conducted random-effects meta-analyses and explored associated factors using multi-step statistical analyses.

**Results:**

We included 35 studies and assessed 2 549 968 participants from 23 countries. The pooled incidence of overall systemic skin reactions was 3.8% (95% confidence interval (CI) = 2.4%-5.5%) with short duration (about one week). Specifically, the pooled incidence rates of rash and urticaria were 3.0% (95% CI = 2.1%-3.9%) and 1.1% (95% CI = 0.7%-1.5%), respectively. For overall local injection site reactions, the pooled incidence was 72.4% (95% CI = 65.7%-78.7%) with short duration (1 to 4.5 days). Except for local pain (72.2%, 95% CI = 65.3%-78.5%), other localized reactions had low incidence, including swelling (13.3%, 95% CI = 9.5%-17.7%), redness (11.5%, 95% CI = 5.7%-19.0%), and erythema (5.8%, 95% CI = 0.7%-15.4%). Geographically, different distribution patterns were observed for these reactions. Regarding associated factors, mRNA vaccines showed lower incidence of urticaria (*P* < 0.001). Asia population showed higher incidence of urticaria (*P* < 0.001). We observed lower incidence rates of overall local injection site reactions and pain among inactivated vaccines (*P* < 0.001). We found no significant difference among reactions between the first and the second dose of vaccines.

**Conclusions:**

We examined the global incidence pattern of common cutaneous reactions related to COVID-19 vaccination and found low incidence and short duration of systemic skin reactions and local injection site reactions (except for pain); discrepancies in these reactions were observed across different vaccine types. The cutaneous side effects related to COVID-19 vaccination do not seem to cause concern.

**Registration:**

PROSPERO: CRD42021258012.

Since first identified in 2019, the coronavirus disease 2019 (COVID-19) rapidly expanded globally and has been declared a pandemic and public health crisis, infecting 603 million and resulting in 6.4 million deaths worldwide, affecting more than 200 countries, according to the World Health Organization (WHO) [1]. For the general population, vaccination is one of the most effective measures for preventing coronavirus infection. Several types of COVID-19 vaccines have been approved worldwide, including mRNA vaccines, adenovirus viral vector (AVV) vaccines, inactivated virus vaccines, and others [2]. They can significantly reduce the risk of severe symptoms, hospitalization, or death from COVID-19 [3]. For example, a two-dose regimen of Pfizer-BioNTech mRNA vaccine conferred 95% protection against COVID-19 in persons aged 16 years or older [4], while the Moderna mRNA vaccine showed 94.1% efficacy at preventing COVID-19 [5].

After the approval of COVID-19 vaccines, as of September 2022, more than 12.5 billion vaccine doses have been administered based on WHO data [1]. During this time, cases of adverse skin reactions to COVID-19 vaccination have been continuously reported [6-8]. As a dermatology research team, we focused on the incidence pattern and factors associated cutaneous reactions following the COVID-19 vaccination. According to clinical trials of mRNA vaccines, local adverse reactions were common (>80%), the most common being localized pain; other localized reactions (e.g. swelling and erythema) had lower incidence (<10%) [5]. Clinical trials of inactivated virus vaccines reported lower incidence of local adverse reaction (about 3%) [9]. For AVV vaccines, the incidence was approximately 50% [10]. While rarely reported in clinical trials [5,9,10], systemic skin responses such as rash have been more commonly reported in real-world studies [11,12]. Furthermore, the factors associated with these cutaneous reactions are another concerned issue [11,13].

As more real-world studies report cutaneous reactions following COVID-19 vaccination, a deeper insight could be gained by summarizing their data through appropriate statistical analysis and exploring associated factors. We aimed to conduct a systematic review and meta-analysis to estimate the global incidence pattern of common cutaneous reactions related to COVID-19 vaccination in real-world settings and to identify factors associated with these reactions.

## METHODS

We performed and reported this study following the Meta-analysis Of Observational Studies in Epidemiology (MOOSE) checklist [14] and registered the protocol in the International Prospective Register of Systematic Reviews (PROSPERO), registration number CRD42021258012.

### Literature search

We searched PubMed, Web of Science, Embase, CNKI and Wanfang from inception to May 13, 2022, without language restrictions. The full search strategies can be found in Table S1 in the **Online Supplementary Document**. We also conducted a manual search using the abovementioned electronic databases and searched the website of *Morbidity and Mortality Weekly Report* [15] for other potentially relevant studies.

### Study selection

We included studies reporting the incidence rates of common cutaneous reactions related to COVID-19 vaccines in real-world settings, including the systematic skin reactions (rash and urticaria) and the local injection site reactions (pain, swelling, redness, and erythema). Incidence refers to the proportion of a population receiving the vaccine that develops cutaneous reactions. The primary outcomes of this study were the overall systematic skin reaction and the overall local injection site reaction; the secondary outcomes were several specific common reported cutaneous reactions, including rash, urticaria, local injection site pain, local injection site swelling, local injection site redness, and local injection site erythema.

We excluded clinical trials, as we were primarily interested in the incidence of cutaneous reactions related to COVID-19 vaccines in real-world settings. We also excluded studies that did not report the exact rate of or did not focus on cutaneous reactions. Additionally, we did not apply restrictions on types or brands of the COVID-19 vaccines. We included conference abstracts with enough information.

For the study selection processes, two researchers independently evaluated the studies in each step; disagreements were solved through group discussions. After deduplication, we screened the titles and abstracts of remaining studies, excluding irrelevant ones, including case reports, reviews, and others. We then evaluated the full texts of remaining studies.

### Data extraction

Two researchers independently extracted information from included studies using a pre-designed standardized data extraction form (Table S2 in the **Online Supplementary Document**), with any disagreements resolved by consensus. This included the following data: basic study information, study population characteristics, information on the COVID-19 vaccine, information of cutaneous reactions related to COVID-19 vaccination, etc. Additionally, we extracted detailed data from studies which reported several findings for population with different characteristics, meaning more than one datapoints could be obtained from one single study.

### Quality assessment

Two researchers independently assessed the methodological quality of included studies using the 11-item quality assessment tool recommended by the Agency for Healthcare Research and Quality (AHRQ) (Table S3 in the **Online Supplementary Document**). One point was given if the item was answered as “Yes”; 0 points were given if the item was answered as “No” or “Unclear”. Totally, a maximum of 11 points could be given for a study, with higher points representing higher quality. The methodological quality of the enrolled studies was categorized as low (0-3 points), medium (4-7 points), and high (8-11 points) [16].

### Statistical analysis

We performed all statistical analyses using Stata Statistical Software and R statistical language. We included datapoints with reports of three common types of vaccines (mRNA vaccine, inactivated virus vaccine, and AVV vaccine) and sample size >100 in the meta-analyses. Additionally, for the overall local injection site reactions, we included studies which reported at least incidence of local injection site pain avoid under-estimating the pooled values. We calculated the pooled estimates for the incidence and the 95% confidence intervals (CIs) using the *metaprop* Stata command to perform meta-analysis of binomial data [17]. The *I*^2^ statistic was calculated to quantify the heterogeneity across studies; the statistical heterogeneity across studies was identified if the *P*-value for Cochran’s Q was <0.05 or the *I*^2^ was >50% [18]. We used a random effects model if we detected heterogeneity; otherwise, we used a fixed effects model. To detect the risk of publication bias, we generated funnel plots and conducted the Egger’s and Begg’s test, considering publication bias if *P* < 0.05 either test [19].

To explore factors associated with cutaneous reactions, we undertook the following multi-step statistical analyses. First, we detected the heterogeneity across studies, as described above. Then, we performed sensitivity analyses by excluding studies one by one and repeating the meta-analysis using the remaining data. Furthermore, we conducted the univariate meta-regression analyses focusing on several available factors, including age groups, ethnic groups, vaccine types, and vaccine doses. Accordingly, we conducted a stratified the analyses by these factors to show detailed incidence rates in each subgroup.

## RESULTS

**Figure 1:**
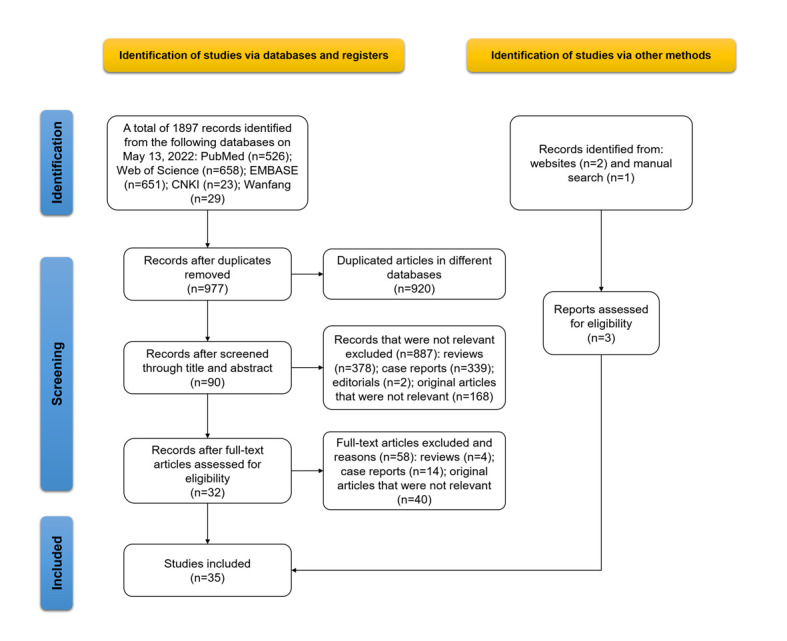
Study selection flowchart.

The search retrieved a total of 1897 records, 920 of which were excluded after deduplication. We excluded 887 records after the title and abstract screening and a further 58 after the full-text review, including reviews (n = 4), case reports (n = 14), and original articles that were not relevant (n = 40). Additionally, three studies were identified through the manual search and website search. Finally, we included 35 studies with 86 datapoints [11,12,20-52]. The study selection process is presented in **Figure 1**.

### Study characteristics and quality assessment

**Figure 2:**
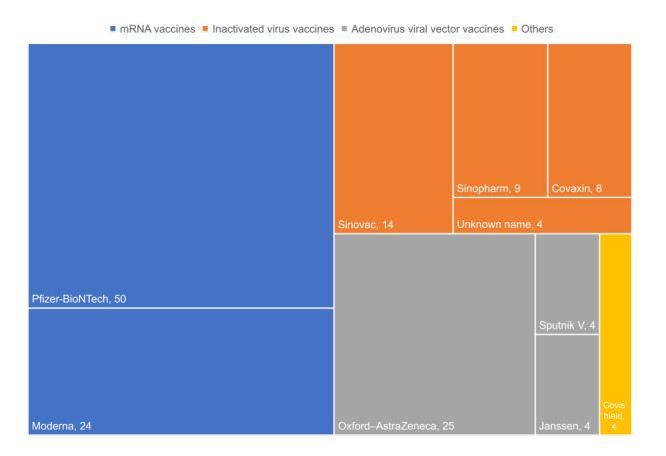
Tree map summarizing the number of datapoints according to types and brands of COVID-19 vaccines. Box sizes indicate the number of datapoints included in the present study.

The included studies’ characteristics are shown in **Table 1**. There were 23 full articles, five letters, three conference abstracts, and four reports/short reports/brief communications. The studies included a total of 2 549 968 participants covering 23 countries. Regarding cutaneous reactions related to COVID-19 vaccination, 32 studies reported local injection site reactions and 21 reported systemic skin reactions. Three most common COVID-19 vaccines were evaluated, including mRNA vaccine, inactivated virus vaccine, and AVV vaccine. As shown in **Figure 2**, Pfizer-BioNTech (BNT162b2) mRNA vaccine was the most studied vaccine, accounting for 50 datapoints, followed by Oxford-AstraZeneca (ChAdOx1 nCoV-19) AVV vaccine (25 datapoints) and Moderna (mRNA-1273) mRNA vaccine (24 datapoints).

**Table 1:** Characteristics of the included studies

References, year of publication (study location)	Vaccine and dose	Study population	Key findings of systemic skin reactions	Key findings of local injection site reactions
Zdanowski et al. [20], 2022 (Poland)	Pfizer-BioNTech (mRNA vaccines); dose 1 & 2	The COVID-19-free group: 145 pregnant (31.4 ± 2.6 y) and 19 non-pregnant (33.1 ± 3.6 y) women health care professionals. The COVID-19-exposed group: 24 pregnant (31.3 ± 1.9 y) and 16 non-pregnant (34.0 ± 4.4 y) women health care professionals.	The COVID-19-free group: the overall systemic skin reactions incidence rate: 1) after the first dose 9.7% (pregnant) and 4.8% (non-pregnant); 2) after the second dose 6.7% (pregnant) and 5.3% (non-pregnant). The COVID-19-exposed group: the overall systemic skin reactions incidence rate: 1) after the first dose 0% (pregnant) and 25.0% (non-pregnant); 2) after the second dose 0% (pregnant) and 13.3% (non-pregnant).	The COVID-19-free group: the overall local injection site reactions incidence rate: 1) after the first dose 94.5% (pregnant) and 100% (non-pregnant); 2) after the second dose 87.7% (pregnant) and 100% (non-pregnant). The COVID-19-exposed group: the overall local injection site reactions incidence rate: 1) after the first dose 100% (pregnant) and 100% (non-pregnant); 2) after the second dose 87.5% (pregnant) and 86.7% (non-pregnant).
Wang et al. [21], 2022 (China)	Sinovac (inactivated virus vaccines); dose 2	60 systemic lupus erythematosus (SLE) patients aged 40.1 ± 12.5 y (3.3% males). 70 rheumatoid arthritis (RA) patients aged 40.7 ± 11.7 y (8.6% males). 35 healthy controls (HCs) aged 39.5 ± 10.2 (8.6% males).	The incidence rates of rash: 3.3% (SLE), 0% (RA), and 0% (HCs). The incidence rates of myalgia: 6.7% (SLE), 4.3% (RA), and 8.6% (HCs).	The incidence rates of local injection site swelling: 1.7% (SLE), 2.9% (RA), and 0% (HCs).
Son et al. [22], 2022 (USA)	Janssen (adenovirus viral vector vaccine), Moderna (mRNA vaccine), and Pfizer-BioNTech (mRNA vaccine); Dose: not reported clearly	256 994 participants (50.0 ± 16.6 y, 25.8% males) using data from the Vaccine Adverse Event Reporting System (VAERS), including: Janssen group: 22 037 participants aged 44.7 ± 15.1 y (33.8% males); Moderna group: 126 838 participants aged 51.5 ± 16.8 y (23.8% males); Pfizer-BioNTech group: 10 8119 participants aged 49.4 ± 16.4 y (26.5% males).	In Janssen group, overall systemic skin reactions incidence rate: 15.3%. In Moderna group, overall systemic skin reactions incidence rate: 12.1%. In Pfizer-BioNTech group, overall systemic skin reactions incidence rate: 15.1%.	Not reported
Rerknimitr et al. [23], 2022 (Thailand)	Sinovac (inactivated virus vaccine) and Oxford–AstraZeneca (adenovirus viral vector vaccine); Dose: 1 & 2	Sinovac group: 29 907 participants aged 34.0 (28.0, 43.5). Oxford-AstraZeneca group: 5322 participants aged 48.0 (35.0, 55.0).	Sinovac group: 1) after the first dose, the median duration of urticaria: 2 d; 2) after the second dose, the median duration of urticaria: 1 d. Oxford-AstraZeneca group: 1) after the first dose, the median duration of urticaria: 4 d; 2) after the second dose, the median duration of urticaria: 3 d.	Sinovac group: the overall cutaneous adverse reactions incidence rate: 1) after the first dose 0.94%; the median duration: 3 d; 2) after the second dose 0.70%. Oxford-AstraZeneca group: the overall cutaneous adverse reactions incidence rate: 1) after the first dose 1.0%; the median duration: 4.5 d; 2) after the second dose 0.52%.
Pourani et al. [24], 2022 (Iran)	Sputnik V (adenovirus viral vector vaccine), Sinopharm (inactivated virus vaccine), Covaxin (inactivated virus vaccine), and Oxford–AstraZeneca (adenovirus viral vector vaccine); Dose: 1 or 2	761 participants (92.9% health care workers) aged 28.1 ± 11.9 y (29.7% males).	In Sputnik V group, the incidence rates: 4.5% (rash) and 2.8% (urticaria). In Sinopharm group, the incidence rates: 3.9% (rash) and 2.5% (urticaria). In Covaxin group, the incidence rates: 5.0% (rash) and 5.0% (urticaria). In Oxford-AstraZeneca group: the incidence rates: 3.3% (rash) and 3.9% (urticaria). On average, the duration of rash and urticaria: 8.79 and 5.48 d	In Sputnik V group, the incidence rates: 12.8% (erythema) and 79.9% (myalgia). In Sinopharm group, the incidence rates: 6.9% (erythema) and 60.6% (myalgia). In Covaxin group, the incidence rates: 5.0% (erythema) and 90.6% (myalgia). In Oxford-AstraZeneca group: the incidence rates: 18.7% (erythema) and 88.0% (myalgia). On average, the duration of erythema: 5.55 d
Ozdede et al. [25], 2022 (Turkey)	Sinovac (inactivated virus vaccine) and Pfizer-BioNTech (mRNA vaccine); Dose 1 or 2	256 patients with Behçet’s syndrome (BS) aged 43.2 ± 10.1 y (62.1% males), 247 with familial Mediterranean fever (FMF) aged 40.0 ± 10.3 y (40.9% males), and 601 with rheumatic diseases (RD) aged 49.3 ± 12.1 y (28.6% males).	Not reported.	In Sinovac group, the incidence rate of overall local injection site reactions: 1.8% (BS), 0% (FMF), 2.6% (RD). In Pfizer-BioNTech group, the incidence rate of overall local injection site reactions: 5.4% (BS), 6.4% (FMF), 7.8% (RD).
Massip et al. [26], 2022 (France)	Moderna (mRNA vaccine) and Oxford–AstraZeneca (adenovirus viral vector vaccine); Dose 1 or 2	70 participants aged 59.0 (37.5-70.0) years (31.4% males).	In Moderna group, the incidence rate of urticaria: 14.3%. In Oxford–AstraZeneca group, the incidence rate of urticaria: 14.3%.	In Moderna group, the incidence rate of overall local injection site reactions: 19.0%. In Oxford-AstraZeneca group, the incidence rate of overall local injection site reactions: 39.2%.
Kitagawa et al. [27], 2022 (Japan)	Pfizer-BioNTech (mRNA vaccine) and Moderna (mRNA vaccine); Dose 1 & 2	Pfizer-BioNTech group: 890 participants (36.4% males). Moderna group: 6401 participants (54.1% males).	Pfizer-BioNTech group: the skin rash incidence rate: 1) after the first dose 2.4%; 2) after the second dose 3.4%. Moderna group: the skin rash incidence rate: 1) after the first dose 5.3%; 2) after the second dose 6.5%. Overall duration: 1-2 d.	Pfizer-BioNTech group: the local injection site pain incidence rate: 1) after the first dose 91.7%; 2) after the second dose 89.1%. Moderna group: the local injection site pain incidence rate: 1) after the first dose 92.9%; 2) after the second dose 93.0%. Overall duration: 1-2 d.
Khan et al. [28], 2022 (Bangladesh)	Oxford–AstraZeneca (adenovirus viral vector vaccine); Dose 1 & 2	293 participants aged 40.3 ± 8.7 y (45.7% males).	The skin rash incidence rate: 1) after the first dose 3.1%; 2) after the second dose 1.0%.	The local injection site pain incidence rate: 1) after the first dose 58.7%; 2) after the second dose 48.5%.
Kamble et al. [29], 2022 (India)	Oxford–AstraZeneca (adenovirus viral vector vaccine); Dose 1	836 health care workers aged 35.8 ± 9.5 y (61.2% males).	Not reported.	Overall local injection site reactions incidence rate: 6.9%.
Durmaz et al. [30], 2022 (Turkey)	Sinovac (inactivated virus vaccine); Dose 1	211 health care workers: 110 males (aged 37.0 ± 13.8 y) and 111 females (aged 38.6 ± 13.3 y).	The incidence rate of urticaria: 5.7%.	The local injection site pain incidence rate: 29.4%.
Du et al. [31], 2022 (China)	Inactivated virus vaccine (nor report the brand name); Dose 1 & 2	72 patients with wheat-dependent exercise-induced anaphylaxis (WDEIA) aged 42.4 (19.0-79.0) years (51.4% males). 730 healthy matched controls aged 40.8 (16.0-76.0) years (53.3% males).	Not reported.	The WDEIA group: the overall local injection site reactions incidence rate: 1) after the first dose 43.1%; 2) after the second dose 35.7%; the local injection site pain incidence rate: 1) after the first dose 41.7%; 2) after the second dose 34.3%; the local injection site redness incidence rate: 1) after the first dose 2.8%; 2) after the second dose 1.4%; the local injection site swelling incidence rate: 1) after the first dose 9.7%; 2) after the second dose 7.1%. The healthy matched controls: the overall local injection site reactions incidence rate: 1) after the first dose 33.8%; 2) after the second dose 27.3%; the local injection site pain incidence rate: 1) after the first dose 30.8%; 2) after the second dose 26.2%; the local injection site redness incidence rate: 1) after the first dose 3.0%; 2) after the second dose 2.6%; the local injection site swelling incidence rate: 1) after the first dose 6.2%; 2) after the second dose 5.9%.
Bawane et al. [32], 2022 (India)	Covishield (virus-like particle vaccine) and Covaxin (inactivated virus vaccine); Dose 1 & 2	1029 health care workers.	Not reported.	The Covishield group: the overall local injection site reactions incidence rate: 1) after the first dose 34.3%; 2) after the second dose 35.3%. The Covaxin group: the overall local injection site reactions incidence rate: 1) after the first dose 60.0%; 2) after the second dose 40.5%; median duration: 3 d (IQR 1-3).
Araminda et al. [33], 2022 (Indonesia)	Oxford–AstraZeneca (adenovirus viral vector vaccine) and Moderna (mRNA vaccine); Dose 1 & 2	406 participants aged 18-30 y (26.6% males), including 205 participants received Oxford–AstraZeneca vaccine and 201 participants had received Moderna vaccine.	Oxford-AstraZeneca group: the skin rash incidence rate: 1) after the first dose 3.9%; 2) after the second dose 1.0%. Moderna group: the skin rash incidence rate: 1) after the first dose 4.0%; 2) after the second dose 6.0%.	Oxford-AstraZeneca group: the local injection site pain incidence rate: 1) after the first dose 82.4%; 2) after the second dose 66.8%. Moderna group: the local injection site pain incidence rate: 1) after the first dose 80.6%; 2) after the second dose 72.1%.
Yang et al. [34], 2021 (China)	Sinopharm (inactivated virus vaccine); Dose 1 & 2	248 162 individuals in a large-scale emergency use.	Not reported.	Overall local injection site reactions incidence rate: 1) after the first dose 0.44%; 2) after the second dose 0.29%.
Tebaa et al. [35], 2021 (Morocco)	Oxford–AstraZeneca (adenovirus viral vector vaccine); Dose: not report clearly	40 health care workers aged 35.4 ± 32.3 y (30.6% males).	Not reported.	The incidence rate of local injection pain: 61.8%.
Shavit et al. [36], 2021 (Israel)	Pfizer-BioNTech (mRNA vaccine); Dose 1	429 adults with wigh allergy risk aged 52 (16) years (29.1% males).	Not reported.	The incidence rates of local injection reactions: 73.5% (injection site pain) and 14.0% (swelling).
Robinson et al. [37], 2021 (USA)	Pfizer-BioNTech (mRNA vaccine) and Moderna (mRNA vaccine); Dose 1 & 2	40 640 health care workers.	Pfizer-BioNTech group: the overall systemic skin reactions incidence rate: 1) after the first dose 1.4%; 2) after the second dose 1.4%; the skin rash incidence rate: 1) after the first dose 1.2%; 2) after the second dose 1.1%; the urticaria incidence rate: 1) after the first dose 0.2%; 2) after the second dose 0.3%. Moderna group: the overall systemic skin reactions incidence rate: 1) after the first dose 2.1%; 2) after the second dose 2.6%; the skin rash incidence rate: 1) after the first dose 1.6%; 2) after the second dose 1.8%; the urticaria incidence rate: 1) after the first dose 0.5%; 2) after the second dose 0.7%.	Not reported.
Riad et al. [38], 2021 (Germany and Czech)	Oxford–AstraZeneca (adenovirus viral vector vaccine); Dose 1	92 health care workers aged 35.4 ± 12.6 y (22.8% males).	The incidence rate of skin rash: 4.3%.	The incidence rates of local injection reactions: 72.8% (injection site pain), 10.9% (swelling), 10.9% (redness).
Pokorna et al. [39], 2021 (Czech Republic)	Pfizer-BioNTech (mRNA vaccine) and Moderna (mRNA vaccine); Dose 1 or 2	539 health care workers aged 22.9 ± 2.1 y (29.3% males).	The incidence rate of skin rash: 0.4%.	The incidence rates of local injection reactions: 91.8% (injection site pain), 17.4% (swelling), 13.4% (redness).
Riad et al. [40], 2021 (Czech Republic)	Pfizer-BioNTech (mRNA vaccine); Dose 1 or 2	874 health care workers aged 42.6 ± 10.5 y (11.4% males).	The incidence rates of systemic skin reactions: 3.2% (rash), 1.1% (urticaria).	The incidence rates of local injection reactions: 83.6% (injection site pain), 23.8% (swelling), 21.4% (redness).
Hockova et al. [41], 2021 (Slovakia)	Pfizer-BioNTech (mRNA vaccine); Dose 2	522 health care workers aged 37.8 ± 11.6 y (23.0% males).	The incidence rates of systemic skin reactions: 1.5% (rash), 2.5% (angioedema).	The incidence rates of local injection reactions: 85.2% (injection site pain), 10.2% (swelling), 8.4% (redness).
Moura et al. [42], 2021 (Portugal)	Pfizer-BioNTech (mRNA vaccine); Dose 1 or 2	3073 health care workers aged 40.2 ± 13.4 y (25.8% males).	The incidence rate of urticaria: 0.2%.	Not reported.
Lim et al. [12], 2021 (Singapore)	Pfizer-BioNTech (mRNA vaccine); Dose 1 & 2	1704 health care workers (age range: 18-80; 21.4% males).	Overall systemic skin reactions incidence rate: 1) after the first dose 2.5%; 2) after the second dose 5.3%.	Overall local injection site reactions incidence rate: 1) after the first dose 57.2%; 2) after the second dose 70.1%.
Klugar et al. [11], 2021 (Germany)	Pfizer-BioNTech (mRNA vaccine), Moderna (mRNA vaccine), and Oxford–AstraZeneca (adenovirus viral vector vaccine); Dose 1 or 2	599 health care workers (median age: 39 y; 27.2% males).	mRNA vaccine group: the incidence rates: 2.5% (skin rash), 0.4% (urticaria). Adenovirus viral vector vaccine group: the incidence rates: 4.0% (skin rash), 1.6% (urticaria).	mRNA vaccine group: the incidence rates: 77.4% (injection site pain), 18.6% (injection site swelling), 10.8% (injection site redness). Adenovirus viral vector vaccine group: the incidence rates: 68.8% (injection site pain), 16.0% (injection site swelling), 8.8% (injection site redness).
Kadali et al. [43], 2021 (USA)	Moderna (mRNA vaccine); Dose 1 or 2	432 health care workers with mean age 43.8 y (10.4% males).	The incidence rate of rash: 13.4%.	The incidence rates of local injection reactions: 94.2% (injection site pain), 15.1% (swelling).
Im JH et al. [44], 2021 (Korea)	Pfizer-BioNTech (mRNA vaccine); Dose 1 & 2	1876 health care workers aged 20–65 y (23.4% males).	The incidence rate of urticaria: 1) after the first dose 2.3%; 2) after the second dose 2.7%.	The local injection site pain incidence rate: 1) after the first dose 84.9%; 2) after the second dose 90.4%. The local injection site redness incidence rate: 1) after the first dose 14.1%; 2) after the second dose 31.9%. The local injection site swelling incidence rate: 1) after the first dose 16.5%; 2) after the second dose 35.5%.
Choi et al. [45], 2021 (Korea)	Pfizer-BioNTech (mRNA vaccine); Dose 1 & 2	638 participants aged 80 (77-83) years (44.4% males).	Not reported.	The overall local injection site reactions incidence rate: 1) after the first dose 50.3%; duration <3 d: 93.8%; 2) after the second dose 45.2%; duration <3 d: 86.6%. The local injection site pain incidence rate: 1) after the first dose 49.4%; duration <3 d: 94.0%; 2) after the second dose 42.8%; duration <3 d: 86.7%. The local injection site swelling incidence rate: 1) after the first dose 3.6%; duration <3 d: 91.3%; 2) after the second dose 5.7%; duration <3 d: 81.8%. The local injection site erythema incidence rate: 1) after the first dose 0.2%; duration <3 d: 100%; 2) after the second dose 1.0%; duration <3 d: 83.3%.
Bookstein Peretz et al. [46], 2021 (Israel)	Pfizer-BioNTech (mRNA vaccine); Dose 1 & 2	390 pregnant (32.5 ± 3.7 y) and 260 non-pregnant (32.4 ± 3.7 y) women.	For the pregnant women, the rash incidence rate: 1) after the first dose 0.8%; 2) after the second dose 1.3%. For the non-pregnant women, the rash incidence rate: 1) after the first dose 0.8%; 2) after the second dose 0.4%.	For the pregnant women, the overall local injection site reactions incidence rate: 1) after the first dose 91.8%; 2) after the second dose 92.3%. For the non-pregnant women, the overall local injection site reactions incidence rate: 1) after the first dose 96.2%; 2) after the second dose 90.4%.
Atteno et al. [47], 2021 (Italy)	Pfizer-BioNTech (mRNA vaccine); Dose 1 & 2	97 participants, including 47 patients with rheumatic diseases (RDs) and 50 healthy controls.	Not reported.	The local injection site pain incidence rate: 1) after the first dose 38.1%; 2) after the second dose 53.6%.
Ali et al. [48], 2021 (USA)	Pfizer-BioNTech (mRNA vaccine) and Moderna (mRNA vaccine); Dose 1 & 2	113 recipients of allogeneic hematopoietic stem cell transplant (HCT) aged 66.5 (22-77) years (69.0% males).	Not reported.	The local injection site pain incidence rate: 1) after the first dose 40.4%; 2) after the second dose 43.8%.
Al Bahrani et al. [49], 2021 (Saudi Arabia)	Oxford–AstraZeneca (adenovirus viral vector vaccine); Dose 1	1592 participants aged 37.4 ± 9.6 y (range 19-83) (81.0% males).	The incidence rate of skin rash: 19.3%.	The local injection site pain incidence rate: 30.5%.
Aga et al. [50], 2021 (Iraq and Jordan)	Pfizer-BioNTech (mRNA vaccine), Oxford–AstraZeneca (adenovirus viral vector vaccine), and Sinopharm (inactivated virus vaccine); Dose 1 & 2	1736 participants aged 49 (IQR 26-74) years (range 18-86) (51.6% males).	Not reported.	Pfizer-BioNTech group: the overall local injection site reactions incidence rate: 1) after the first dose 25.0%; 2) after the second dose 13.0%. Oxford–AstraZeneca group: the overall local injection site reactions incidence rate: 1) after the first dose 37.5%; 2) after the second dose 1.0%. Sinopharm group: the overall local injection site reactions incidence rate: 1) after the first dose 8.0%; 2) after the second dose 12.0%.
Shay et al. [51], 2021 (USA)	Janssen (adenovirus viral vector vaccine); Single-dose needed	338 700 Janssen COVID-19 vaccine recipients who completed at least one v-safe survey (active monitoring system).	Not reported.	The overall local injection site reactions incidence rate: 61%.
Gee et al. [52], 2021 (USA)	Pfizer-BioNTech (mRNA vaccine) and Moderna (mRNA vaccine); Dose 1 or 2	1 602 065 vaccine recipients who completed at least one v-safe survey (active monitoring system).	Not reported.	The incidence rates: 70.9% (injection site pain), 10.8% (injection site swelling).

AVV vaccine – adenovirus viral vector vaccine, AZ – Oxford–AstraZeneca, BS – Behcet’s syndrome, FMF – familial Mediterranean fever, HCs – healthy controls, Pfizer – Pfizer-BioNTech vaccine, RA – rheumatoid arthritis; RD – rheumatic disease, SLE – systemic lupus erythematosus, WDEIA – wheat-dependent exercise-induced anaphylaxis, d – days, y – years

We evaluated the included studies’ methodological quality using the AHRQ tool as shown in Figure S1 in the **Online Supplementary Document**. The total score of these studies ranged from 2 to 7 and 62.9% (n = 22) of them were considered moderate quality.

### Incidence pattern and factors associated with systemic skin reactions

**Figure 3:**
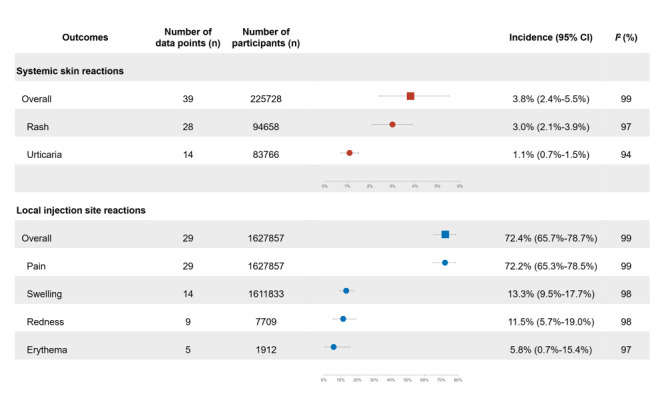
Incidence of common cutaneous reactions related to COVID-19 vaccination.

**Figure 4:**
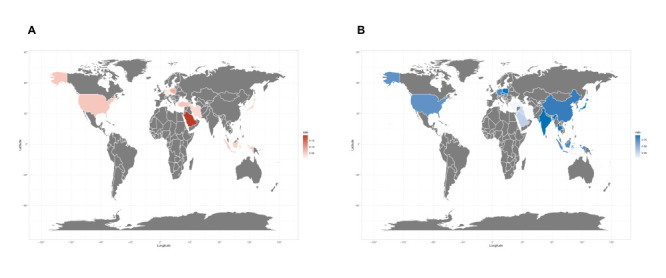
Global maps for incidence of overall cutaneous reactions related to COVID-19 vaccination. **Panel A:** Global map for incidence of overall systematic skin reactions. **Panel B**: Global map for incidence of overall local injection site reactions.

According to the random-effects meta-analysis, the pooled incidence of overall systemic skin reactions was 3.8% (95% CI = 2.4%-5.5%) (**Figure 3** and Figure S2 in the **Online Supplementary Document**). Specifically, the pooled incidence rates of rash and urticaria were 3.0% (95% CI = 2.1%-3.9%) and 1.1% (95% CI = 0.7%-1.5%), respectively (**Figure 3** and Figure S3-4 in the **Online Supplementary Document** The global distribution of overall systemic skin reactions was shown in **Figure 4**, panel A and Table S4 in the **Online Supplementary Document**. The highest and the lowest ones were overserved in Saudi Arabia (19.3%) and Portugal (0.2%). Similarly, for the local injection site reactions, the three types of vaccines showed different distribution patterns of overall systemic skin reactions (Figure S5-7 in the **Online Supplementary Document**).

**Figure 5:**
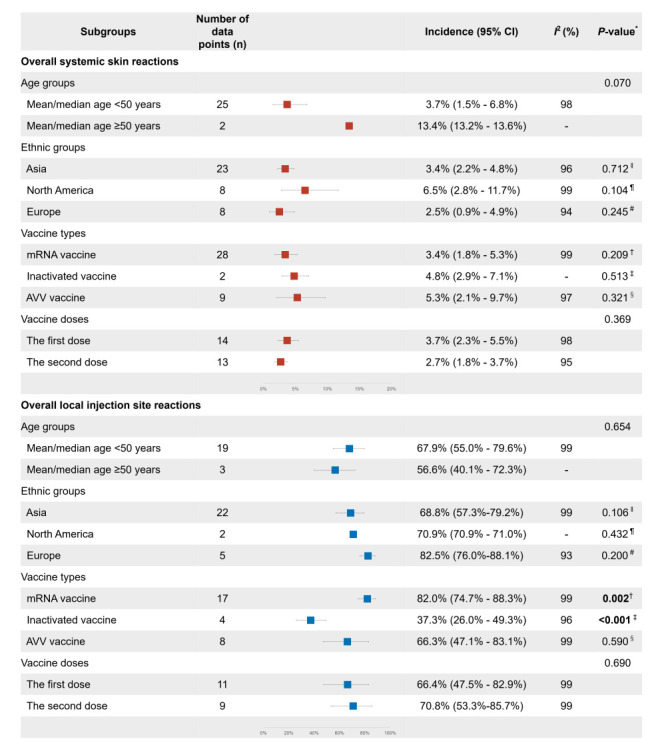
Meta-regression analyses and subgroup analyses of overall cutaneous reactions. *Univariate meta-regression analyses. †mRNA vaccine vs non-mRNA vaccine. ‡Inactivated vaccine vs non-inactivated vaccine. § AVV vaccine vs non-AVV vaccine. ‖Asia group vs non-Asia groups. ¶North America group vs non-North America groups. #Europe group vs non-Europe groups. AVV vaccine – adenovirus viral vector vaccine, CI – confidence interval.

**Figure 6:**
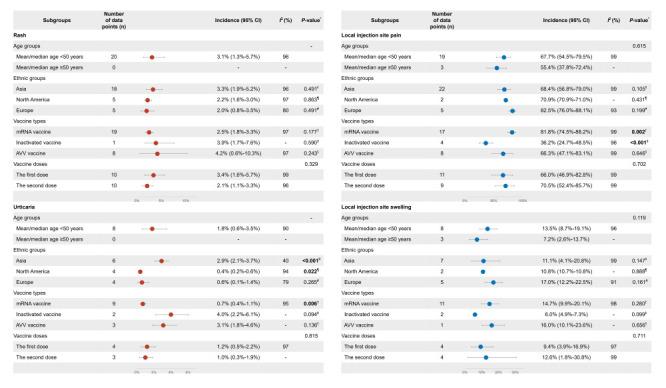
Meta-regression analyses and subgroup analyses of specific cutaneous reactions. *Univariate meta-regression analyses. †mRNA vaccine vs non-mRNA vaccine. ‡Inactivated vaccine vs non-inactivated vaccine. §AVV vaccine vs non-AVV vaccine. ‖Asia group vs non-Asia groups. ¶North America group vs non-North America groups. #Europe group vs non-Europe groups. AVV vaccine – adenovirus viral vector vaccine, CI – confidence interval.

We conducted further analyses to detect potential factors associated with systemic skin reactions. As shown in **Figure 3**, we noted high levels of heterogeneity across studies (*I*^2^>50%). Based on the leave-one-out sensitivity analyses, no single study significantly affected the initial findings (Figure S8-10 in the **Online Supplementary Document**). For the overall systemic skin reactions and rash, the results of meta-regression and subgroup analyses suggested that there were no significant contributions to heterogeneity in the ethnic groups (*P* > 0.05), vaccine types (*P* > 0.05), and vaccine doses (*P* > 0.05) (**Figure 5** and **Figure 6**). Despite a small number of studies (n = 2), we observed higher incidence of overall systemic skin reactions among the group with mean/median age ≥50 years (13.4%, 95% CI = 13.2%-13.6% vs 3.7%, 95% CI = 1.5%-6.8%; *P* = 0.070) (**Figure 5**). For urticaria, mRNA vaccines had significant lower incidence rates (*P* = 0.006); the incidence rates for mRNA vaccines, inactivated vaccines, and AVV vaccines were 0.7% (95% CI = 0.4%-1.1%), 4.0% (95% CI = 2.2%-6.1%), and 3.1% (95% CI = 1.8%-4.6%), respectively (**Figure 6**). Regarding ethnic groups, the Asian population (2.9%, 95% CI = 2.1%-3.7%) showed significant higher incidence of urticaria (*P* < 0.001) compared with North America population (0.4%, 95% CI = 0.2%-0.6%) and European population (0.6%, 95% CI = 0.1%-1.4%) (**Figure 6**).

Regarding publication bias, the non-symmetric funnel plots and the Egger’s test (*P* < 0.001) indicated publication bias for overall systemic skin reactions (Figure S11 in the **Online Supplementary Document**). However, for rash and urticaria, we found no evidence of publication bias through visualization of funnel plots and the results of Egger’s and Begg’s tests (Figure S12-13 in the **Online Supplementary Document**).

Regarding the duration of systemic skin reactions, major findings [22,23,26] were summarized in the Table S6 in the **Online Supplementary Document**. We reported on inconsistent results. The median duration for rash ranged from one to 8.79 days; the median duration for urticaria ranged from one to 5.48 days.

### Incidence pattern and factors associated with local injection site reactions

For overall local injection site reactions, according to the random-effects meta-analysis, we estimated the pooled incidence rate to be 72.4% (95% CI = 65.7%-78.7%) (**Figure 3** and Figure S14 in the **Online Supplementary Document**). The most common specific local injection site reaction was local injection site pain (72.2%, 95% CI = 65.3%-78.5%). Other localized reactions had lower incidence, including swelling (13.3%, 95% CI = 9.5%-17.7%), redness (11.5%, 95% CI = 5.7%-19.0%), and erythema (5.8%, 95% CI = 0.7%-15.4%), respectively (**Figure 3** and Figure S15-18 in the **Online Supplementary Document**). Geographically, the incidence of local injection site reactions varied considerably; the highest and the lowest ones were observed in India (93.1%) and Thailand (0.7%) (**Figure 4**, panel B and Table S5 in the **Online Supplementary Document**). We observed different distribution patterns for the three types of vaccines (Figure S19-21 in the **Online Supplementary Document**).

We conducted further analyses to detect potential factors associated with local injection site reactions. First, we observed statistical heterogeneity across studies (all *I*^2^>50%) (**Figure 3**). Further leave-one-out sensitivity analyses indicated that the pooled estimation of local injection site reactions incidence was robust and that no single study excessively influenced the results (Figure S22-26 in the **Online Supplementary Document**). We then performed meta-regression analyses focusing on multiple factors and subgroup analyses (**Figure 5**). We found that vaccine type contributed to heterogeneities across studies (*P* < 0.05). We did not find significant heterogeneity in incidence of overall local injection site reactions among different age groups (*P* > 0.05), ethnic groups (*P* > 0.05), and vaccine doses (*P* > 0.05). However, for mRNA vaccines, inactivated vaccines, and AVV vaccines, the incidence rates were 82.0% (95% CI = 74.7%-88.3%), 37.3% (95% CI = 26.0%-49.3%), and 66.3% (95% CI = 47.1%-83.1%) respectively; significant lower incidence was seen in the inactivated vaccine group (*P* < 0.001) (**Figure 5**). Additionally, we did meta-regression analyses and subgroup analyses for specific local injection site reactions, including pain and swelling; given the limited datapoints and relatively lower incidence, we did not conduct these analyses for redness and erythema. Similarly, we found significantly lower incidence of local injection site pain in the inactivated vaccine group (*P* < 0.001) (**Figure 6**). For swelling, we observed marginally significant difference due to limited datapoints (**Figure 6**).

Regarding publication bias for overall local injection site reactions and pain, swelling, redness, and erythema, the funnel plot distributions were not generally symmetric (Figure S27-31 in the **Online Supplementary Document**). Based on the Egger’s test and Begg’s test, we detected potential publication bias for all above-mentioned local injection site reactions.

We summarized major findings of the cutaneous reactions’ duration in Table S6 in the **Online Supplementary Document**. Five studies [23,24,27,32,45] reported the duration of local injection site reactions, with observed discrepancies across studies. Overall, the median duration ranged from one day to 4.5 days [23,27,32]; more than 80% of participants showed duration of <3 days [44].

## DISCUSSION

Based on our analyses of 2.55 million participants, we found low incidence and short duration of systemic skin reactions (rash, among others) and local injection site reactions (except for localized pain). Geographically, we observed different global distribution patterns for these reactions. We identified different incidence of reactions for different types of vaccines; mRNA vaccines showed significant lower incidence of urticaria. We also observed lower incidence rates of overall local injection site reactions and pain in the inactivated vaccines. However, we found similar incidence of these reactions after the first and second dose of vaccines.

As suggested by previous studies, rash and urticaria have been the most studied systemic skin reactions related to COVID-19 vaccines [31]. Moreover, local injection site reactions were one of the most prevalent reactions [53,54]. After vaccination, the human body generates an immune response; meanwhile, the inflammation in the muscle at the injection site may occur [53]. These responses can contribute to the adverse skin reactions [54]. We found that the incidence rates of these systemic reactions were very low and that related concerns were unnecessary. Although the incidence of this localized pain seemed high, we found that the duration of these skin reactions was short, i.e. within five days on average for localized reactions, while systemic skin reactions lasted up to about one weeks. Except for local pain, other localized reactions showed lower incidence (95% CI = 5.8-13.3%). Furthermore, Qaderi et al [54] reported that most COVID-19 vaccine-induced skin reactions were self-limiting and needed little or no therapeutic intervention. Therefore, concerns about adverse skin reactions should not be a reason to avoid vaccination.

Concerning pre-COVID-19 reaction levels, especially the systematic skin reactions (rash and urticaria), we searched data from the Global Burden of Disease (GBD) study [55], which includes 369 diseases and injuries in 204 countries or regions around the world. As shown in the GBD official website [56], the incidence rates of atopic dermatitis (a surrogate disease of rash, as rash was not included in GBD study) in 2018 ranged from 0.11% to 0.71% in different countries, which was much lower than the rash incidence (3.0%, 95% CI = 2.1%-3.9%) after COVID-19 vaccines in our study. Interestingly, for urticaria, the 2018 incidence rates in different countries ranged from 0.83% to 2.72%, which seem similar to the incidence (1.1%, 95% CI = 0.7%-1.5%) in our study. However, this was a preliminary comparison and further investigation is needed, such as the causal inference analysis for COVID-19 vaccines and these skin reactions.

We found discrepancies in these reactions across different vaccine types. We observed a significantly lower incidence of urticaria in mRNA vaccines, while we did not identify such discrepancy in rash. Similarly, another recent published study [56] performed subgroup analysis by vaccine type, suggesting lower incidence of cutaneous adverse reactions in mRNA vaccines. We also noted varying patterns in incidence of urticaria in different ethnic groups and we found a higher incidence of urticaria in Asia compared with North America and Europe. A recently published study [57] reported similar findings. However, different COVID-19 vaccines were utilized in different countries. The COVID-19 vaccines that are most frequently used in the US are mRNA vaccines and AVV vaccines; the inactivated vaccines are more often used in China. For our analysis of urticaria, the utilization rates of mRNA vaccines were 33.3% (n = 2/6) in Asian, 100% (n = 4/4) in North America, and 75% (n = 3/4) in Europe, respectively. Therefore, the discrepancy could be explained by the lower use of mRNA vaccines in Asian countries in our analyses.

We also explored factors associated with local injection site reactions. We noted that mRNA vaccines had higher incidence rate of local injection site reactions, compared with the inactivated vaccines and AVV vaccines. Similar findings have also been reported elsewhere [13,54]. Currently, mRNA vaccines are the most widely used vaccines. They can direct human cells to make a viral spike protein and evoke an immune response by applying a synthetic mRNA that encodes a viral spike protein [58]. The Pfizer-BioNTech (BNT162b2) mRNA vaccine and Moderna (mRNA-1273) mRNA vaccine are two licensed mRNA vaccines and have both shown high protective efficacy (>90%) [4,5]. For the design and packaging of vaccines, both of BNT162b2 and mRNA-1273 contain polyethylene glycol (PEG) in their composition [59]. However, studies on the role of such excipients of COVID-19 vaccines in the onset of cutaneous reactions are limited and further investigation is required.

Whether vaccine recipients of different ages have different incidence of adverse reactions after COVID-19 vaccines is a key topic of concern. A recent pooled analysis [54] showed that local injection site reactions are more prevalent in the younger population compared the older people. Similarly, significantly higher rates of local injection site pain were reported in younger group [36]. We observed higher incidence of overall systemic skin reactions among the participants with mean/median age ≥50 years (*P* = 0.070). This may be due to the small number of studies available for such subgroup analyses; there were only two datapoints enrolling participants with a mean/median age ≥50 years. Moreover, for local injection site reactions, the number of studies with mean/median age ≥50 years was small (n = 3). Further studies are needed to address this issue.

Risk of the second dose of vaccines is another issue of concern. We found similar levels of multiple cutaneous reactions for the first and the second dose, in line with the findings regarding systemic skin reactions reported by Washrawirul et al [60]. Likewise, another study [61] focusing on a population experiencing an allergic reaction to the first dose found that the rates of a second reaction with severe immediate and non-severe symptoms were 4.94% (95% CI = 0.93%-22.28%) and 9.54% (95% CI = 2.18%-33.34%), respectively. These findings demonstrate that a second dose of COVID-19 vaccines should not be consider a risk factor for adverse skin reactions.

Notably, since we focused on real-world settings, we did not include clinical trials in our analyses. The common local reactions related to COVID-19 vaccination have also been assessed in randomized controlled trials (RCTs). In the phase 3 trial of Moderna (mRNA-1273) mRNA vaccine, the vaccine group showed overall local adverse reaction rate of 84.2%; similar to our findings, the most common one was local injection site pain (83.7%) and other localized reactions had lower incidence, including swelling (6.1%) and erythema (2.8%) [5]. Different results for other types of vaccines were reported in RCTs. For inactivated virus vaccines, the incidence of overall local adverse reaction and local pain were reported to be approximately 3%; the incidence of swelling and erythema were reported to be <1% [9]. For AVV vaccine, the phase 3 trial found that the incidence of overall local adverse reaction was about 50%; similar patterns were seen that local pain (about 50%) was the most common one and other localized reactions had lower incidence (about 5% for erythema and swelling) [10]. The discrepancy between real-world studies and RCTs may result from different participant inclusion and exclusion criteria. Regarding systemic reactions, RCTs mainly focused on headache, fever, fatigue, etc. other than systemic skin responses [5,9,10]. Thus, real-world studies could provide better understanding of systemic reactions incidence pattern following COVID-19 vaccination.

### Strengths and limitations

To the best of our knowledge, our study is the most up-to-date and comprehensive systematic review and meta-analysis to evaluate the global incidence pattern of common cutaneous reactions related to COVID-19 vaccination and is the first to extensively explore possible factors associated these reactions in real-world settings. The included studies covered a total of 2.55 million participants from 23 countries. Furthermore, our study has important public health implications for COVID-19 prevention policies. First, we summarized the global data for safety of multiple COVID-19 vaccines and explore possible factors associated these cutaneous reactions, which have been hot topics currently. Second, our findings provide specific support for promoting vaccination in terms of concerns about cutaneous adverse reactions. Based on our findings, public health department could widely disseminate the information on cutaneous adverse reactions to the general population to eliminate concerns to increase vaccination rates.

This study has several limitations. First, we observed substantial heterogeneity across studies, which is a common phenomenon in meta-analysis focusing on rates [62-64]. Accordingly, we conducted the meta-analysis by using random-effects model for data synthesis to estimate the pooled incidence rates. Second, based on our current analyses, we identified potential publication bias for overall systematic skin reactions, local injection site pain, etc. The phenomenon could be explained by the fact that only a part of studies has been published since the approval of COVID-19 vaccines. Accordingly, cautious interpretation is required when extrapolating results to the entire general population. Third, we cannot conduct some subgroup analyses due to lack of eligible original studies. For example, we were not able to evaluate whether the high allergy risk was a potentially associated factor; the number of studies focused on aged people was limited and further investigations are needed. Our systematic review and meta-analysis depend on the primary data; despite the best efforts, subtle methodological issues might have caused deviations of unknown direction and magnitude.

## CONCLUSIONS

We reported the global incidence pattern of common cutaneous reactions related to COVID-19 vaccination and found low incidence and short duration of systemic skin reactions and local injection site reactions (except for pain); we observed discrepancies in these reactions across different vaccine types. Our findings offer comprehensive information, which could be disseminated to eliminate unnecessary concerns and promote COVID-19 vaccination.

## Additional material


Online Supplementary Document
