# Supplementary material for: Global incidence pattern and factors associated with common cutaneous reactions related to COVID-19 vaccination of 2.55 million participants in real-world settings: A systematic review and meta-analysis
Source: J Glob Health. 2023 Feb 10;13:06008. doi: 10.7189/jogh.13.06008 (PMC9910561; doi:10.7189/jogh.13.06008)
Supplement: Online Supplementary Document [file jogh-13-06008-s001.pdf]

ONLINE SUPPLEMENTARY DOCUMENT

Title: Global incidence pattern and factors associated with common cutaneous reactions related to COVID-19 vaccination of 2.55 million participants in real-world settings: a systematic review and meta-analysis

Authors: Yang Guo, Xue-Shan Cao, Hua-Tong Yang, Meng-Ge Zhou, Bo Yu

Table S1. Literature search strategy

Database	Search strategy
PubMed	(((vaccines[Mesh]) OR vaccine[Title/Abstract] OR vaccination [Title/Abstract]) AND ((COVID-19[Mesh]) OR (SARS-CoV-2[Mesh]) OR "Severe Acute Respiratory Syndrome Coronavirus 2"[Title/Abstract] OR (COVID-19[Title/Abstract]) OR (COVID[Title/Abstract]) OR (SARS-CoV-2[Title/Abstract]) OR coronavirus[Title/Abstract]) OR (CoronaVac[Title/Abstract]) OR (COVID-19 Vaccines[Mesh]))) AND (cutaneous[Title/Abstract] OR skin[Title/Abstract] OR rash[Title/Abstract] OR eczema[Title/Abstract])
Web of Science	(TS=(vaccine) OR TS=(vaccination)) AND (TS=(COVID-19) OR TS=(SARS-CoV-2) OR TS=("Severe Acute Respiratory Syndrome Coronavirus 2") OR TS=(coronavirus)) AND (TS=(cutaneous) OR TS=(skin) OR TS=(rash) OR TS=(eczema))
EMBASE	('vaccine'/exp OR 'vaccine':ti,ab,kw OR 'vaccination':ti,ab,kw) AND ('covid 19'/exp OR 'sars cov 2'/exp OR 'COVID-19':ti,ab,kw OR 'COVID':ti,ab,kw OR 'SARS-CoV-2':ti,ab,kw OR 'coronavirus':ti,ab,kw) AND ('cutaneous':ti,ab,kw OR 'skin':ti,ab,kw OR 'rash':ti,ab,kw OR 'eczema':ti,ab,kw)
CNKI	(AB %'vaccine') and (AB %'COVID-19') and (AB %'skin')<br>(Chinese)
Wanfang	(AB: 'vaccine') and (AB: 'COVID-19') and (AB: 'skin')<br>(Chinese)

Table S2. Data extraction form

I. Basic information of included studies

- Authors: ________________________________
- Publication year: __________________________
- Journal: _________________________________
- Title: ___________________________________
- Country/Region: __________________________

II. Characteristics of study population

- Age: _____________________________________
- Male (%): ________________________________
- Description of study population (e.g. healthcare professionals):

____

- Sample size: ___________________________________

III. Information of the COVID-19 vaccine

- Name of the vaccine: ____________________________
- Dose:

□ dose 1

□ dose 2

□ _________

- Type of vaccine:

□mRNA vaccines

□Inactivated virus vaccines

□Adenovirus viral vector vaccines

□ ________________________

IV. Information of cutaneous reactions related to COVID-19 vaccination

➢ Key findings of systematic skin reaction

- Overall description: ____________________

- Rate of specific reactions:

□ Rash: ______(%)

□ Urticaria: ______(%)

□ Others: ______

➢ Key findings of local injection site reactions

- Overall description: ____________________

- Rate of specific reactions:

□ Local pain: ______(%)

□ Local swelling: ______(%)

□ Local redness: ______(%)

□ Local erythema: ______(%)

□ Others: ______

V. Information of data extraction

Reviewer name: ________________________

Date of data extraction: __________________

Table S3. AHRQ tool for cross-sectional/prevalence study

Item	Yes	No	Unclear
1) Define the source of information (survey, record review)			
2) List inclusion and exclusion criteria for exposed and unexposed subjects (cases and controls) or refer to previous publications			
3) Indicate time period used for identifying patients			
4) Indicate whether or not subjects were consecutive if not population-based			
5) Indicate if evaluators of subjective components of study were masked to other aspects of the status of the participants			
6) Describe any assessments undertaken for quality assurance purposes (e.g., test/retest of primary outcome measurements)			
7) Explain any patient exclusions from analysis			
8) Describe how confounding was assessed and/or controlled.			
9) If applicable, explain how missing data were handled in the analysis			
10) Summarize patient response rates and completeness of data collection			
11) Clarify what follow-up, if any, was expected and the percentage of patients for which incomplete data or follow-up was obtained			

AHRQ: Agency for Healthcare Research and Quality

https://www.ncbi.nlm.nih.gov/books/NBK35156/

Table S4. Incidence of overall systemic skin reactions among different regions

Region	Incidence	Vaccine type
Saudi Arabia	19.3%	AVV vaccine
USA	15.3%	AVV vaccine
Poland	8.3%	mRNA vaccine
Turkey	5.7%	inactivated vaccine
USA	5.6%	mRNA vaccine
Indonesia	4.9%	mRNA vaccine
Japan	4.4%	mRNA vaccine
Iran	4.1%	AVV vaccine
Germany	4.0%	AVV vaccine
Iran	3.9%	inactivated vaccine
Singapore	3.7%	mRNA vaccine
Germany	2.5%	mRNA vaccine
South Korea	2.5%	mRNA vaccine
Indonesia	2.2%	AVV vaccine
Bangladesh	1.9%	AVV vaccine
Czech Republic	1.8%	mRNA vaccine
Slovakia	1.5%	mRNA vaccine
Israel	0.8%	mRNA vaccine
Portugal	0.2%	mRNA vaccine

AVV vaccine, adenovirus viral vector vaccine.

Table S5. Incidence of overall local injection site reactions among different regions

Region	Incidence	Vaccine type
India	93.1%	inactivated vaccine
Japan	92.0%	mRNA vaccine
Poland	91.9%	mRNA vaccine
Thailand	91.4%	inactivated vaccine
Israel	89.7%	mRNA vaccine
Czech Republic	87.0%	mRNA vaccine
Iran	85.3%	AVV vaccine
Slovakia	85.2%	mRNA vaccine
China	84.2%	inactivated vaccine
Germany	77.4%	mRNA vaccine
Indonesia	76.5%	mRNA vaccine
Indonesia	75.0%	AVV vaccine
Iraq	73.5%	inactivated vaccine
Jordan	73.5%	inactivated vaccine
USA	70.9%	mRNA vaccine
South Korea	69.8%	mRNA vaccine
Germany	68.8%	AVV vaccine
Singapore	63.8%	mRNA vaccine
USA	61.0%	AVV vaccine
Bangladesh	53.6%	AVV vaccine
Iraq	37.5%	AVV vaccine
Jordan	37.5%	AVV vaccine
Turkey	32.5%	inactivated vaccine
Saudi Arabia	30.5%	AVV vaccine
Iraq	25.0%	mRNA vaccine
Jordan	25.0%	mRNA vaccine
India	6.9%	AVV vaccine
Turkey	6.7%	mRNA vaccine
Iran	5.4%	inactivated vaccine
Thailand	0.7%	AVV vaccine

AVV vaccine, adenovirus viral vector vaccine.

Table S6. Summary of the cutaneous reactions' duration

References (study location)	Vaccine and dose	Study population	Systemic skin reactions	Local injection site reactions
Rerknimitr P et al, 2022 (Thailand)	Sinovac (inactivated virus vaccine) and Oxford–AstraZeneca (adenovirus viral vector vaccine); Dose: 1 & 2	Sinovac group: 29907 participants aged 34.0 (28.0, 43.5)<br>Oxford–AstraZeneca group: 5322 participants aged 48.0 (35.0, 55.0)	Sinovac group:<br>1) after the first dose, the median duration of urticaria: 2 days; 2) after the second dose, the median duration of urticaria: 1 day<br>Oxford–AstraZeneca group:<br>1) after the first dose, the median duration of urticaria: 4 days; 2) after the second dose, the median duration of urticaria: 3 days	The overall cutaneous adverse reactions:<br>Sinovac group: after the first dose, the median duration: 3 days;<br>Oxford–AstraZeneca group: after the first dose, the median duration: 4.5 days
Pourani MR et al, 2022 (Iran)	Sputnik V (adenovirus viral vector vaccine), Sinopharm (inactivated virus vaccine), Covaxin (inactivated virus vaccine), and Oxford–AstraZeneca (adenovirus viral vector vaccine); Dose: 1 or 2	761 participants (92.9% healthcare workers) aged 28.1 ± 11.9 years (29.7% males)	On average, the duration of rash and urticaria: 8.79 and 5.48 days	On average, the duration of erythema: 5.55 days
Kitagawa H et al, 2022 (Japan)	Pfizer-BioNTech (mRNA vaccine) and Moderna (mRNA vaccine); Dose 1 & 2	Pfizer-BioNTech group: 890 participants (36.4% males)<br>Moderna group: 6401 participants (54.1% males)	Duration of overall systemic skin reactions: 1-2 days	Duration of overall local injection site reactions: 1-2 days
Bawane J et al, 2022 (India)	Covishield (virus-like particle vaccine) and Covaxin (inactivated virus vaccine); Dose 1 & 2	1029 healthcare workers	Not reported	The Covaxin group, overall local injection site reactions after the second dose, the median duration: 3 days (IQR 1-3)
Choi YY et al, 2021 (Korea)	Pfizer-BioNTech (mRNA vaccine); Dose 1 & 2	638 participants aged 80 (77-83) years (44.4% males)	Not reported	The overall local injection site reactions: 1) after the first dose, duration < 3 days: 93.8%; 2) after the second dose, duration < 3 days: 86.6%<br>The local injection site pain: 1) after the first dose, duration < 3 days: 94.0%; 2) after the second dose, duration < 3 days: 86.7%<br>The local injection site swelling: 1) after the first dose, duration < 3 days: 91.3%; 2) after the second dose, duration < 3 days: 81.8%<br>The local injection site erythema: 1) after the first dose, duration < 3 days: 100%; 2) after the second dose, duration < 3 days: 83.3%

1	2	3	4	5	6	7	8	9	10	11	Total score	Study	Type
Yes	Yes	Yes	Unclear	Unclear	Yes	Yes	No	No	No	No	5	Zdanowski W et al, 2022	Full article
Yes	Yes	Yes	Unclear	Unclear	No	No	No	No	No	No	3	Wang P et al, 2022	
Yes	Yes	Yes	Unclear	Unclear	Yes	Yes	No	Yes	Yes	No	7	Son C-S et al, 2022	
Yes	No	Yes	Yes	Unclear	Yes	Yes	No	No	Yes	No	6	Ozdede A et al, 2022	
Yes	No	Yes	Unclear	Unclear	Yes	Yes	No	No	No	No	4	Kitagawa H et al, 2022	
Yes	No	Yes	Unclear	Unclear	No	No	No	No	Yes	No	3	Khan MK et al, 2022	
Yes	Yes	Yes	Unclear	Unclear	Yes	Yes	No	No	Yes	No	6	Kamble B et al, 2022	
Yes	Yes	Yes	Unclear	Unclear	Yes	No	No	No	No	No	4	Du Z et al, 2022	
Yes	Yes	Yes	Unclear	Unclear	No	Yes	No	No	Yes	No	5	Araminda GN et al, 2022	
Yes	Yes	Yes	Unclear	Unclear	Yes	Yes	No	No	No	No	5	Yang Z et al, 2021	
Yes	Yes	Yes	Unclear	Unclear	Yes	No	No	No	No	No	4	Shavit R et al, 2021	
Yes	Yes	Yes	Unclear	Unclear	Yes	Yes	No	No	Yes	No	6	Riad A et al, 2021 (Vaccines)	
Yes	No	Yes	Unclear	Unclear	Yes	Yes	No	No	Yes	No	5	Riad A, Pokorna A, et al, 2021 (Pharmaceuticals)	
Yes	Yes	Yes	Unclear	Unclear	Yes	Yes	No	No	Yes	No	6	Riad A et al, 2021 (J Clin Med)	
Yes	Yes	Yes	Unclear	Unclear	Yes	Yes	No	No	Yes	No	6	Riad A, Hockova B, et al, 2021 (Pharmaceuticals)	
Yes	No	Yes	Unclear	Unclear	No	Yes	No	Yes	Yes	No	5	Lim SM et al, 2021	
Yes	Yes	Yes	Unclear	Unclear	Yes	Yes	No	No	Yes	No	6	Klugar M et al, 2021	
Yes	Yes	Yes	Unclear	Unclear	Yes	No	No	No	No	No	4	Kadali RAK et al, 2021	
Yes	No	Yes	Unclear	Unclear	Yes	No	No	No	No	No	3	Choi YY et al, 2021	
Yes	No	Yes	Unclear	Unclear	Yes	Yes	No	No	Yes	No	5	Bookstein Peretz S et al, 2021	
Yes	Yes	Yes	Unclear	Unclear	Yes	No	No	No	No	No	4	Ali H et al, 2021	
Yes	Yes	Yes	Unclear	Unclear	Yes	No	No	No	No	No	4	Al Bahrani S et al, 2021	
Yes	Yes	Yes	Unclear	Unclear	Yes	No	No	No	No	No	4	Aga QAAK et al, 2021	
Yes	Yes	No	Unclear	Unclear	Yes	No	No	No	No	No	3	Rerknimitr P et al, 2022	Letter
Yes	No	Yes	Unclear	Unclear	Yes	No	No	No	No	No	3	Massip E et al, 2022	
Yes	No	Yes	Unclear	Unclear	Yes	No	No	No	No	No	3	Durmaz K et al, 2022	
Yes	No	Yes	Unclear	Unclear	Yes	No	No	No	No	No	3	Bawane J et al, 2022	
Yes	No	Yes	Unclear	Unclear	Yes	No	No	No	No	No	3	Robinson LB et al, 2021	
Yes	No	Yes	Unclear	Unclear	Yes	No	No	No	No	No	3	Tebaa A et al, 2021	Abstract
Yes	No	Yes	Unclear	Unclear	Yes	No	No	No	No	No	3	Moura AL et al, 2021	
Yes	Yes	No	Unclear	Unclear	No	No	No	No	No	No	2	Atteno M et al, 2021	
Yes	No	Yes	Unclear	Unclear	Yes	Yes	No	No	Yes	No	5	Pourani MR et al, 2022	Others
Yes	No	Yes	Unclear	Unclear	No	No	No	No	No	No	2	Shay DK et al, 2021	
Yes	No	Yes	Unclear	Unclear	No	No	No	No	No	No	2	Gee J et al, 2021	
Yes	Yes	Yes	Unclear	Unclear	Yes	Yes	No	No	Yes	No	6	Im JH et al, 2021	

Figure S1. Quality assessment of included studies using the AHRQ tool

AHRQ: Agency for Healthcare Research and Quality; others (type): reports/ short reports/ brief communication

1. Define the source of information (survey, record review);

2 List inclusion and exclusion criteria for exposed and unexposed subjects (cases and controls) or refer to previous publications;

3. Indicate time period used for identifying patients;

4. Indicate whether or not subjects were consecutive if not population-based;

5. Indicate if evaluators of subjective components of study were masked to other aspects of the status of the participants;

6. Describe any assessments undertaken for quality assurance purposes (e.g., test/retest of primary outcome measurements);

7. Explain any patient exclusions from analysis;

8. Describe how confounding was assessed and/or controlled;

9. If applicable, explain how missing data were handled in the analysis;

10. Summarize patient response rates and completeness of data collection;

11. Clarify what follow-up, if any, was expected and the percentage of patients for which incomplete data or follow-up was obtained.

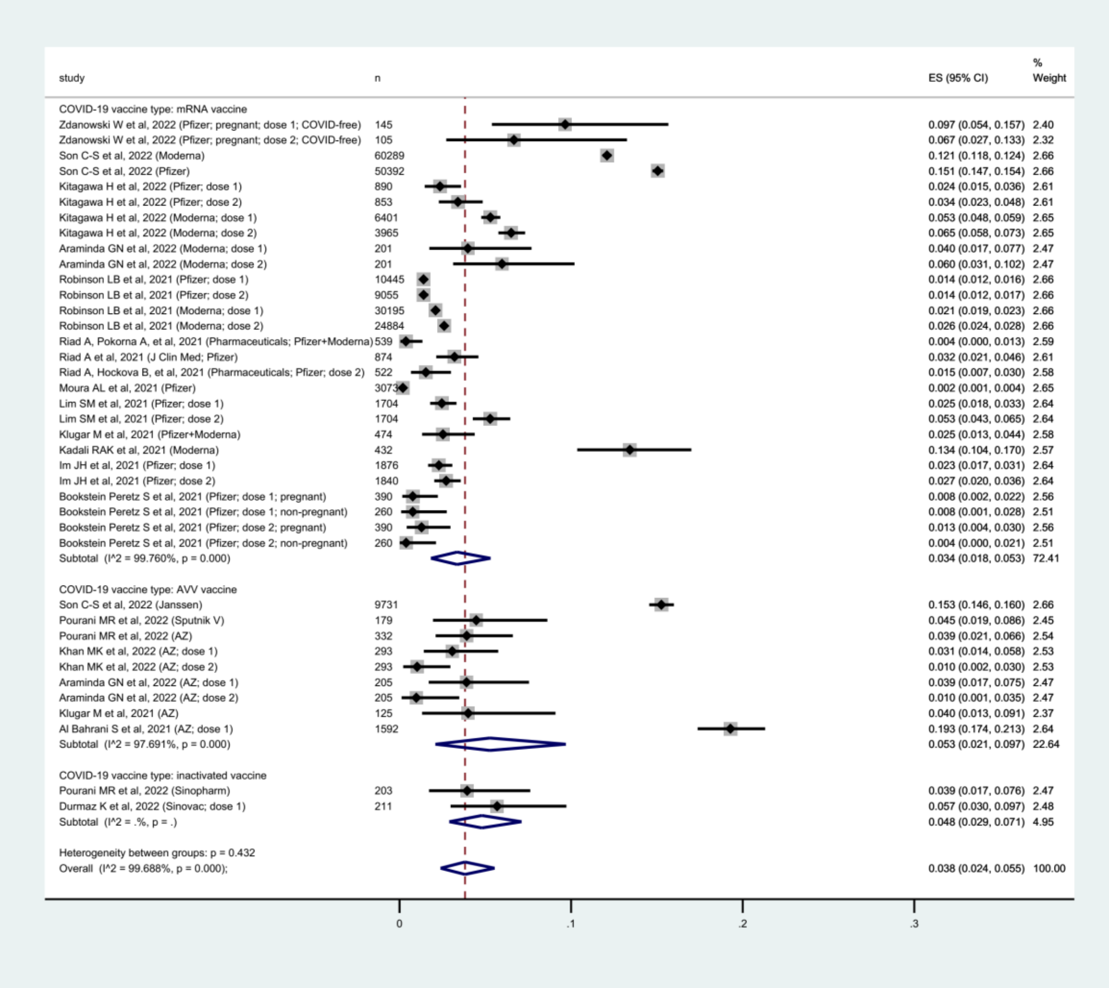

Figure S2. Incidence of overall systematic skin reactions for different types of COVID-19 vaccines

AVV vaccine, adenovirus viral vector vaccine.

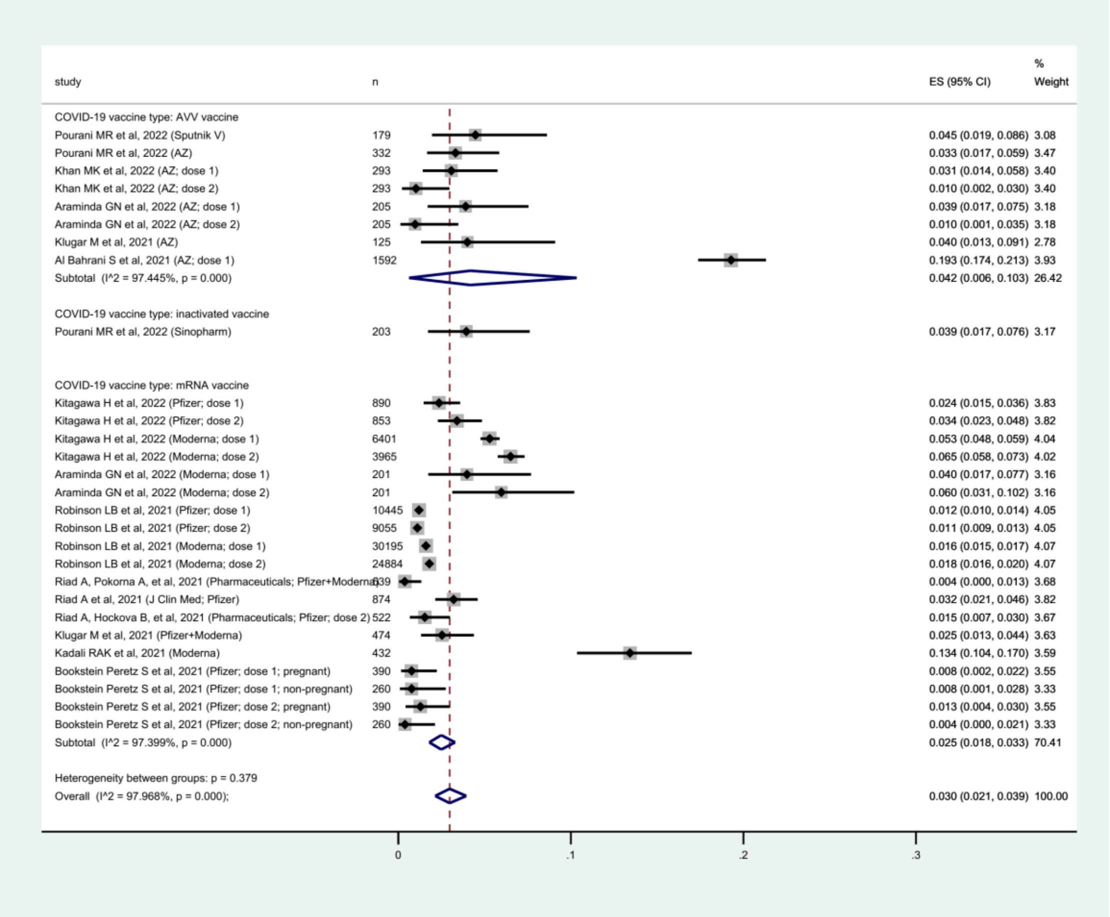

Figure S3. Incidence of rash for different types of COVID-19 vaccines

AVV vaccine, adenovirus viral vector vaccine.

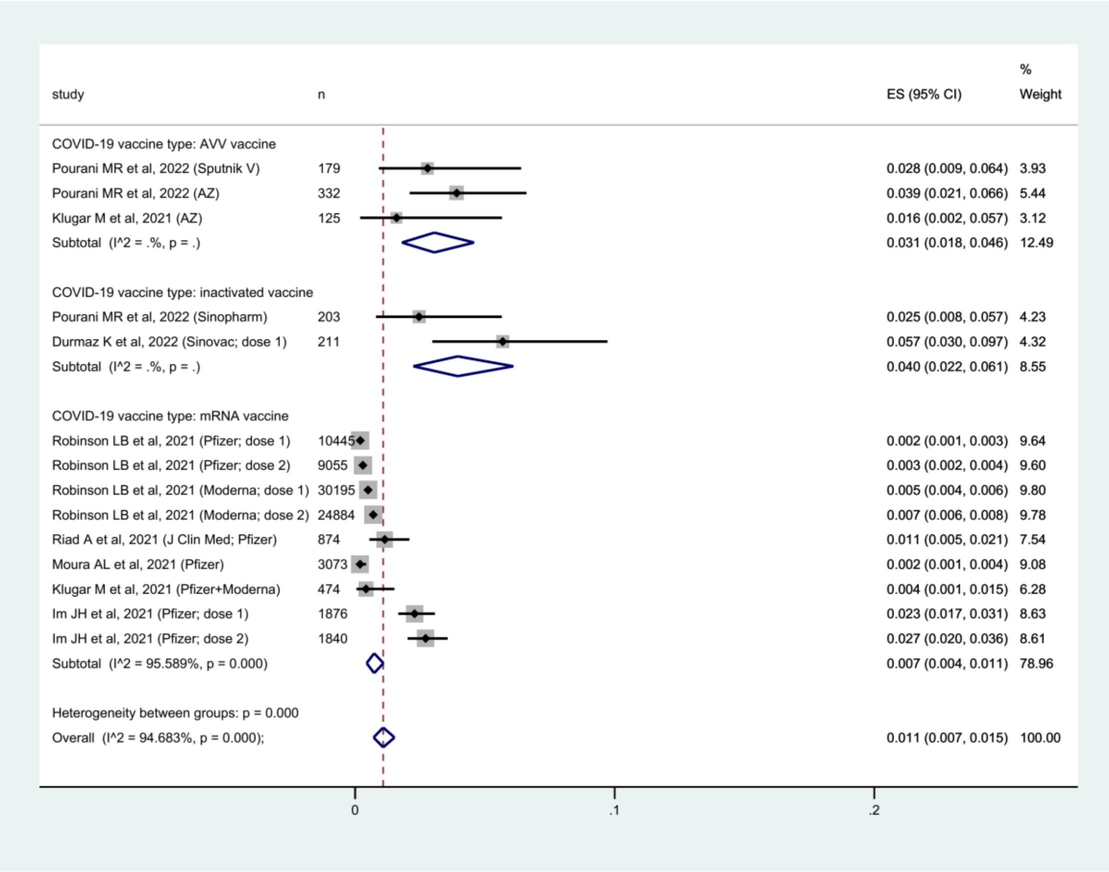

Figure S4. Incidence of urticaria for different types of COVID-19 vaccines

AVV vaccine, adenovirus viral vector vaccine.

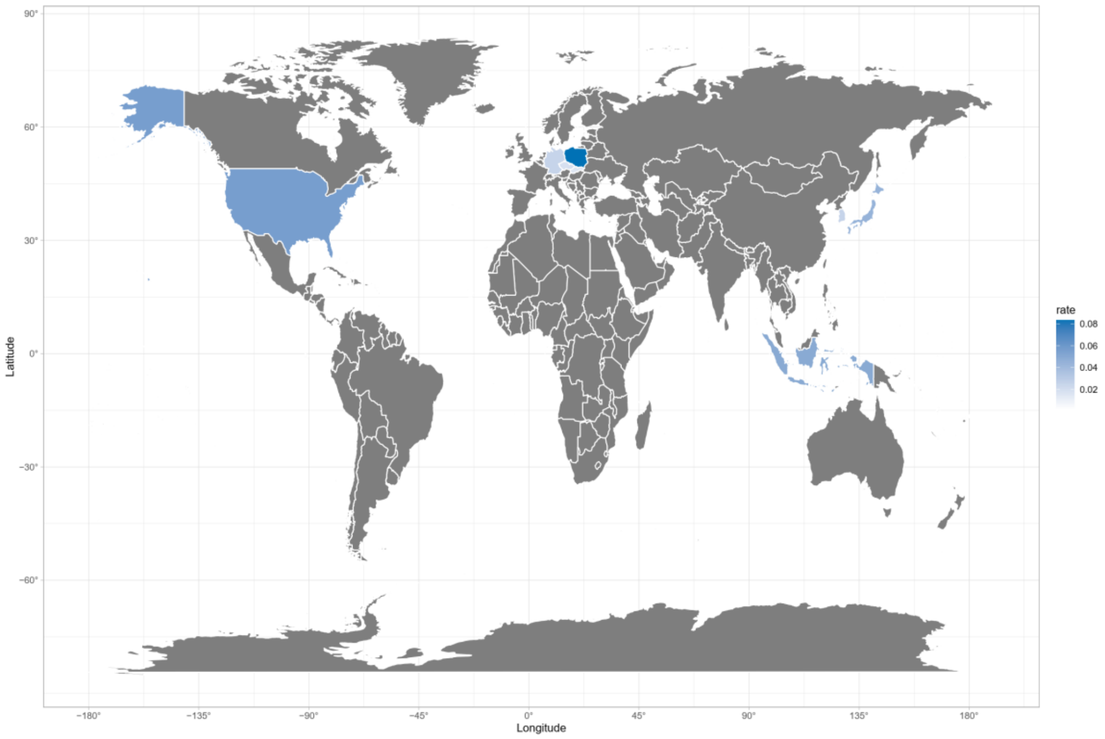

Figure S5. Global map for incidence of overall systematic skin reactions related to mRNA vaccines

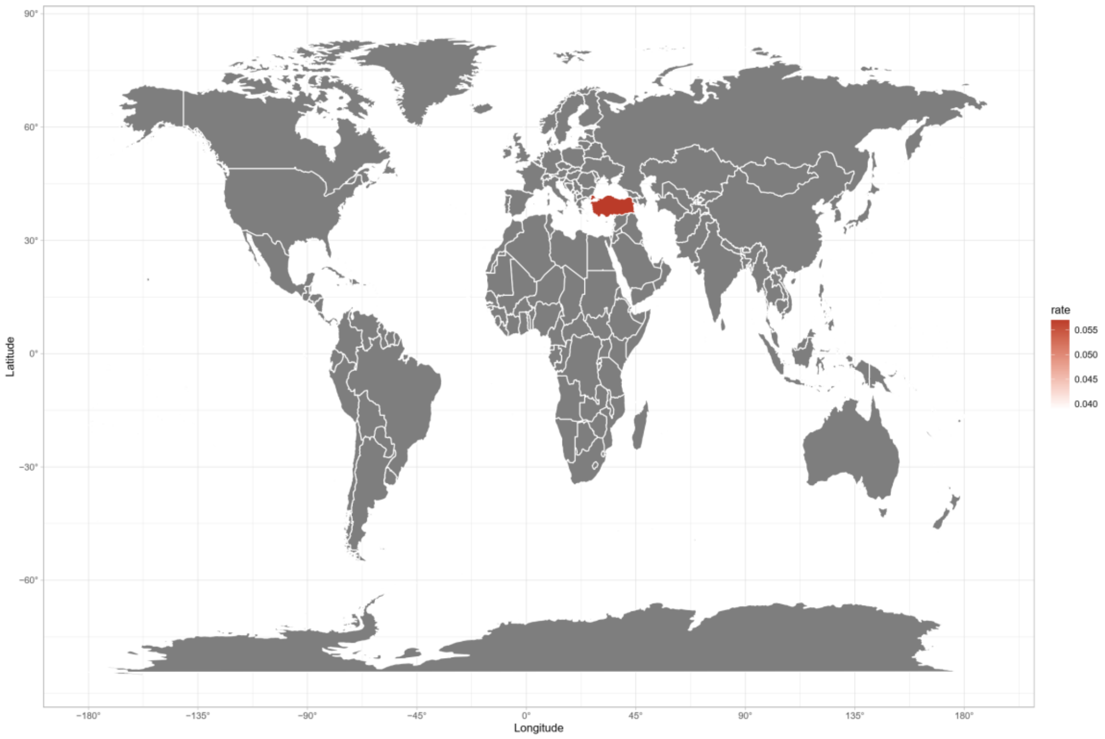

Figure S6. Global map for incidence of overall systematic skin reactions related to inactivated virus vaccines

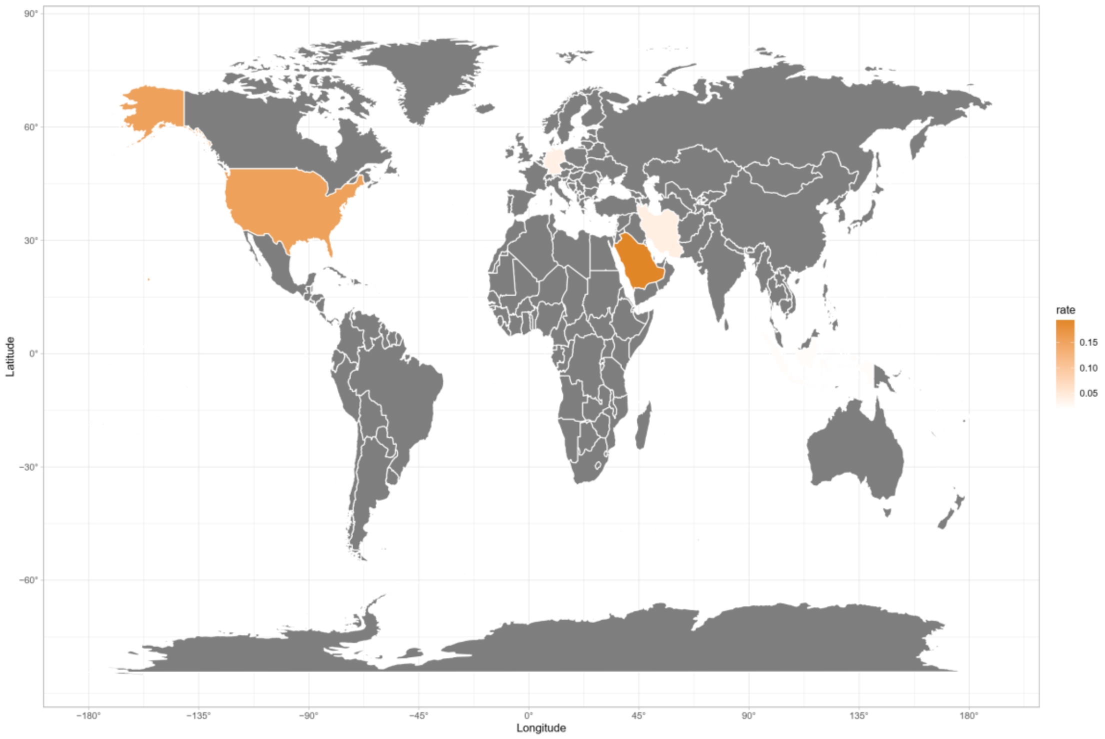

Figure S7. Global map for incidence of overall systematic skin reactions related to adenovirus viral vector vaccines

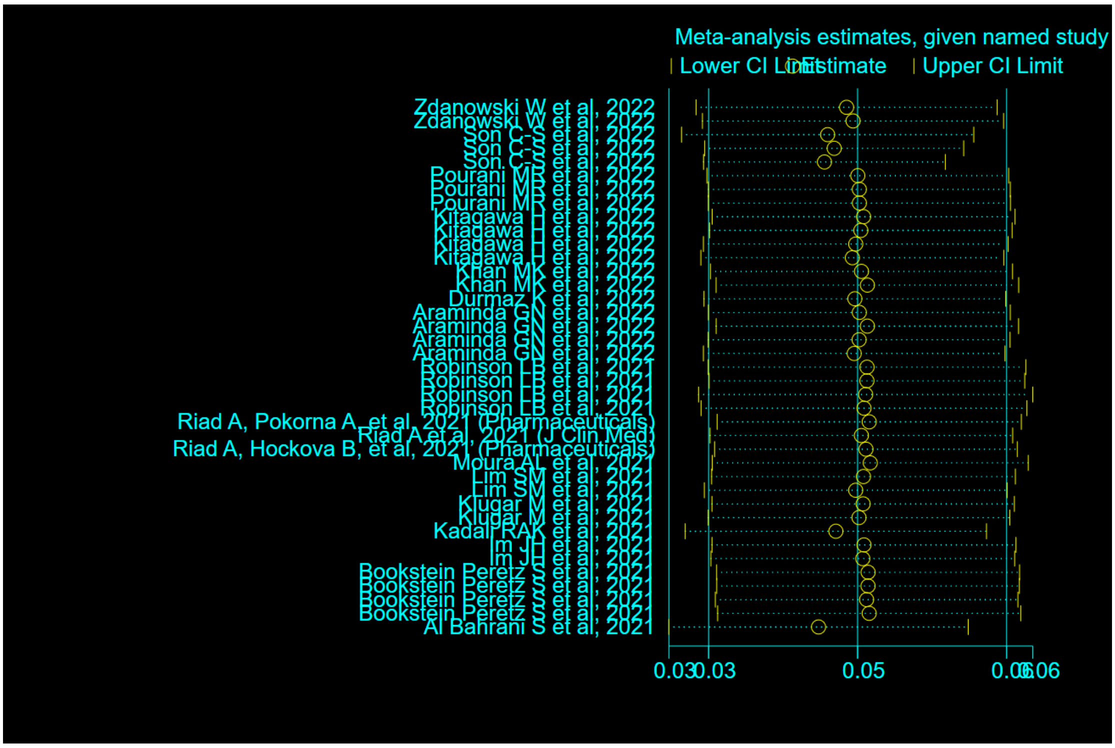

Figure S8. Sensitivity analysis for overall systematic skin reactions

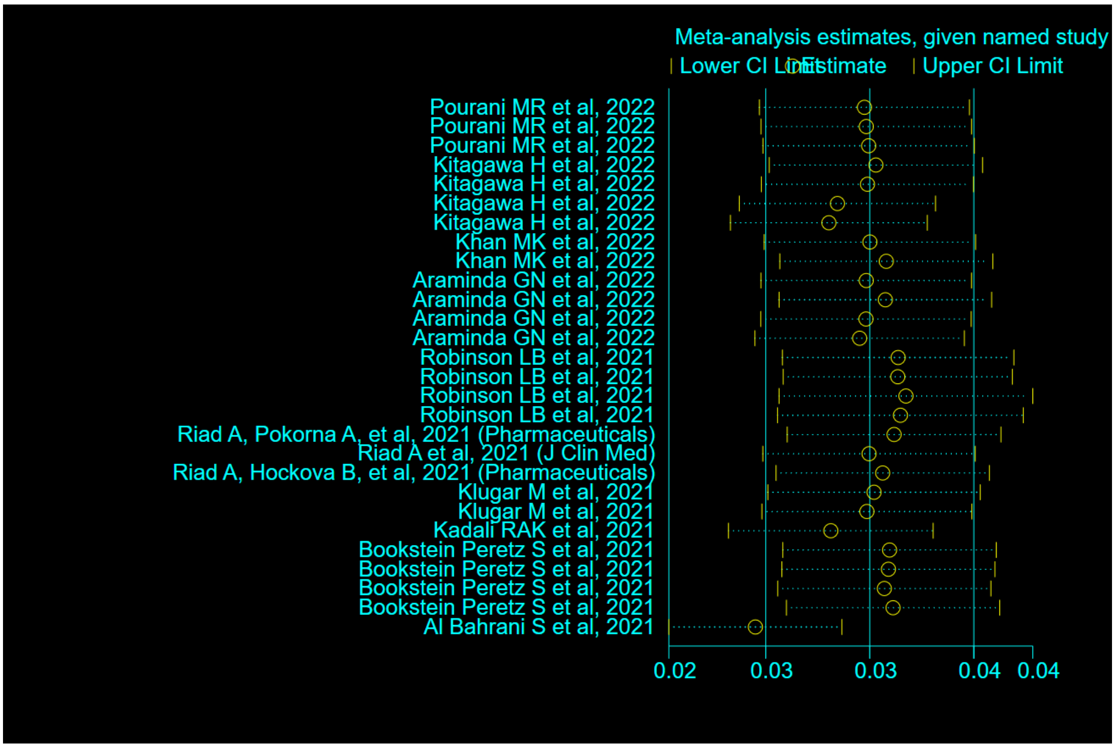

Figure S9. Sensitivity analysis for rash

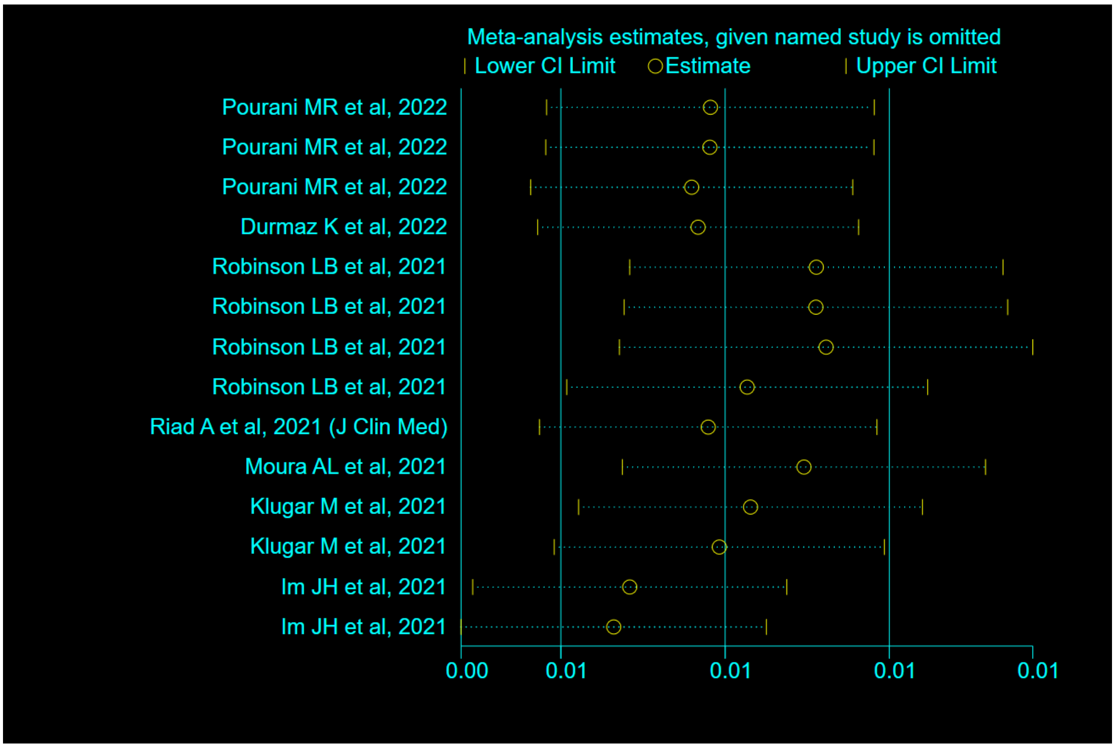

Figure S10. Sensitivity analysis for urticaria

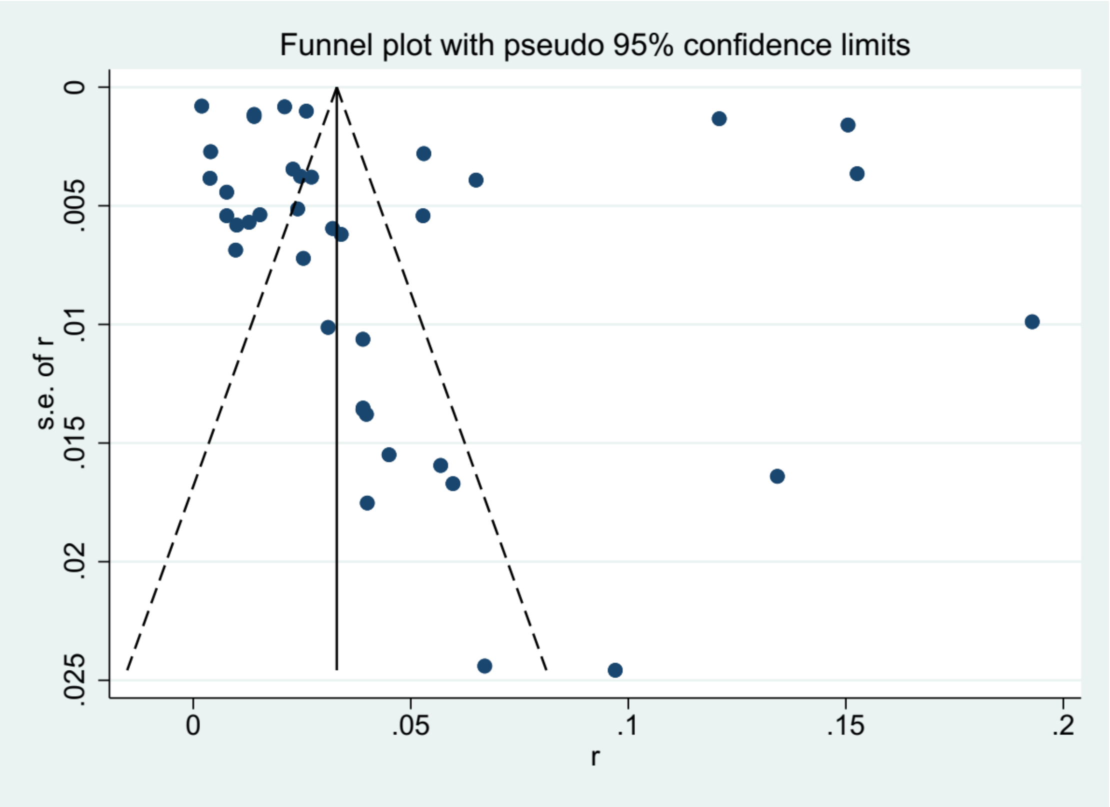

Figure S11. Funnel plots for overall systematic skin reactions

P value for Egger's test <0.001and *P* value for Begg's test =0.179.

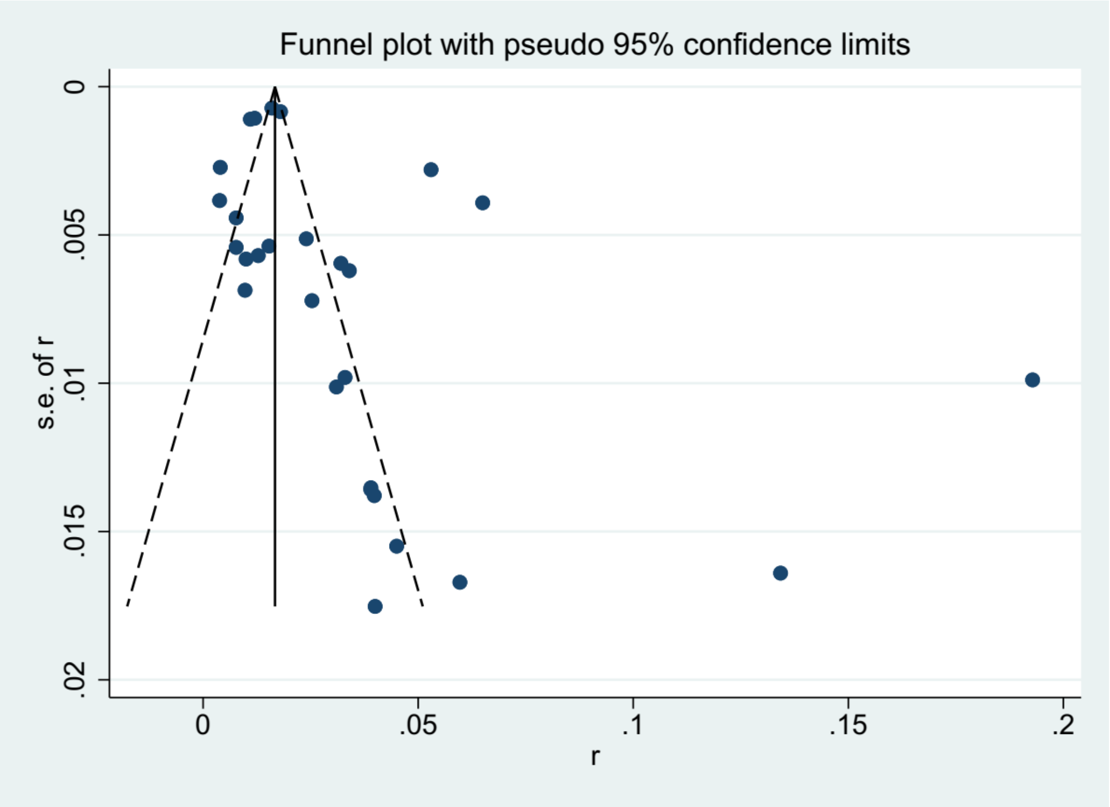

Figure S12. Funnel plots for rash

P value for Egger's test = 0.731 and *P* value for Begg's test = 0.284

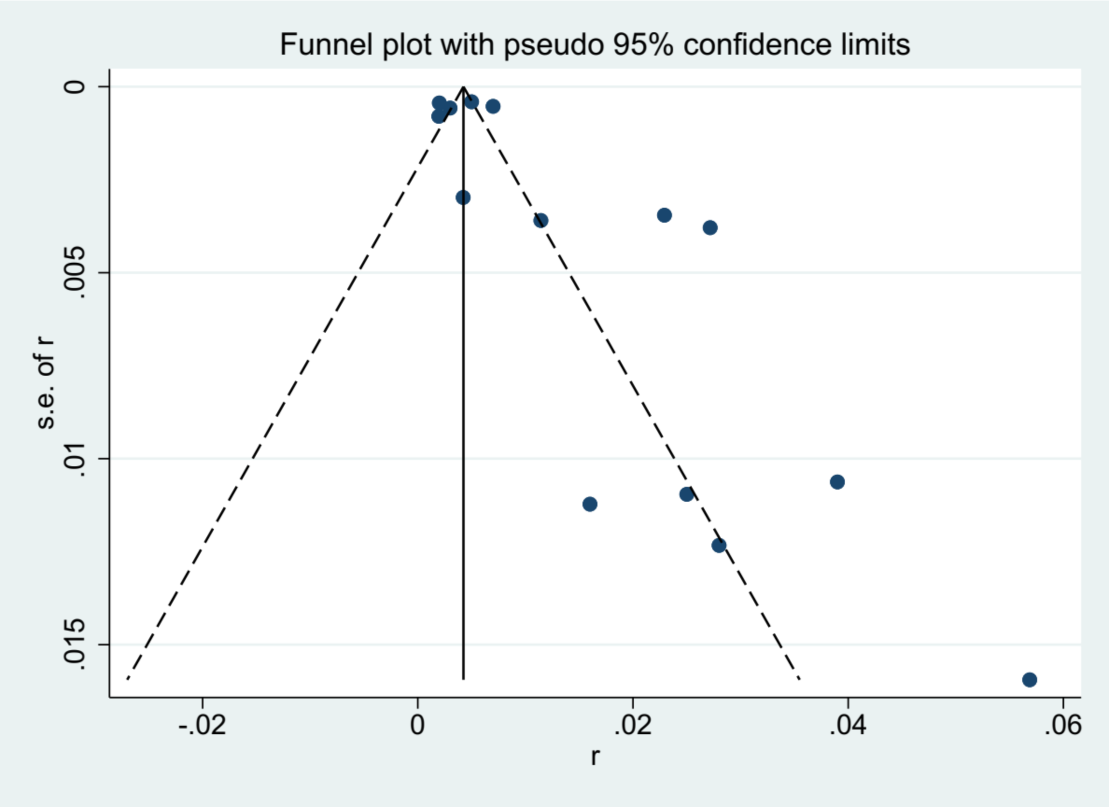

Figure S13. Funnel plots for urticaria

P value for Egger's test = 0.341 and *P* value for Begg's test = 0.956

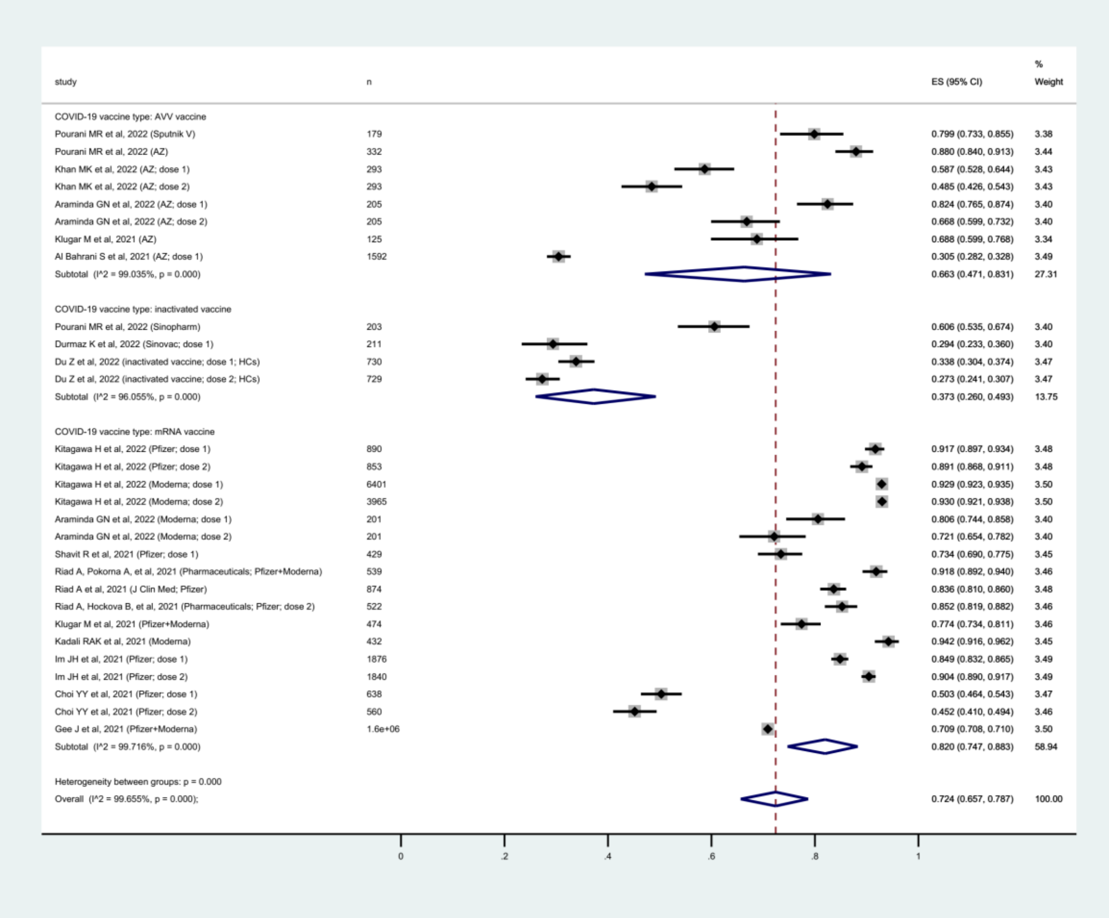

Figure S14. Incidence of overall local injection site reactions for different types of COVID-19 vaccines

AVV vaccine, adenovirus viral vector vaccine.

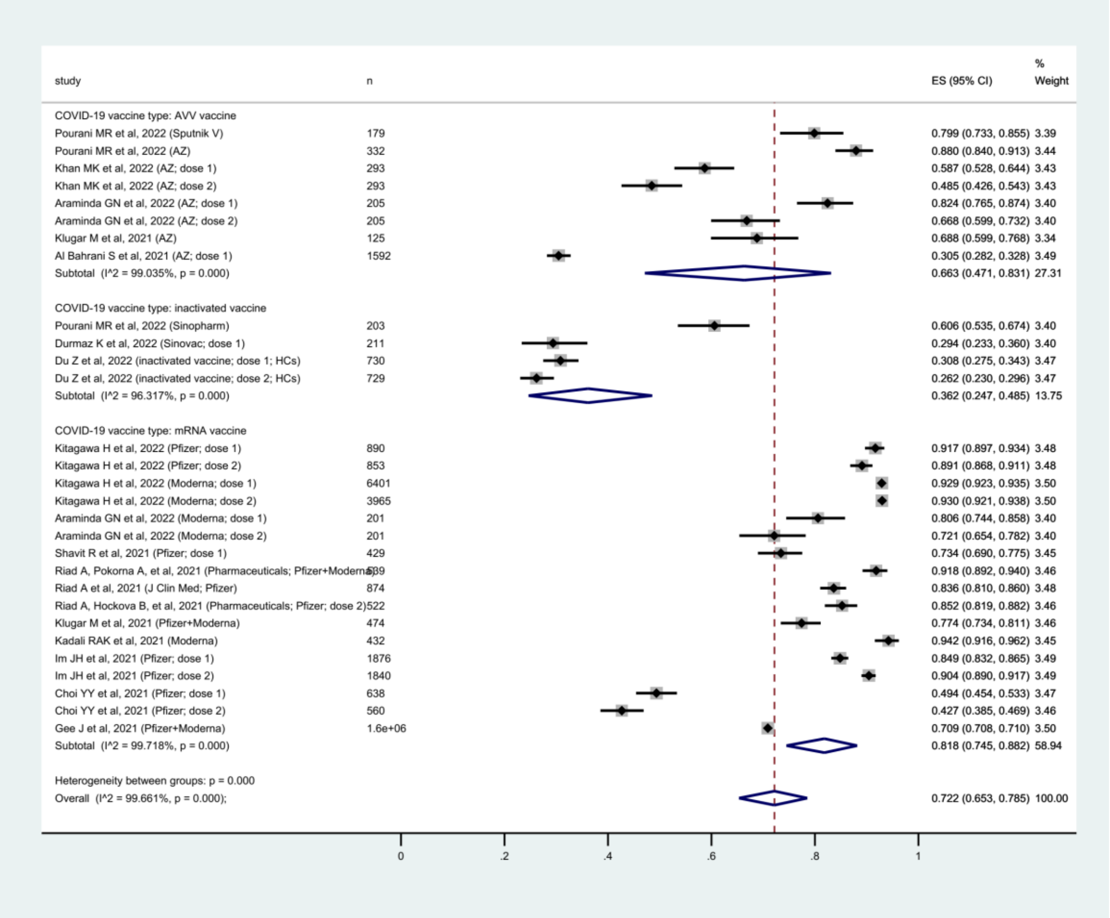

Figure S15. Incidence of local injection site pain for different types of COVID-19 vaccines

AVV vaccine, adenovirus viral vector vaccine.

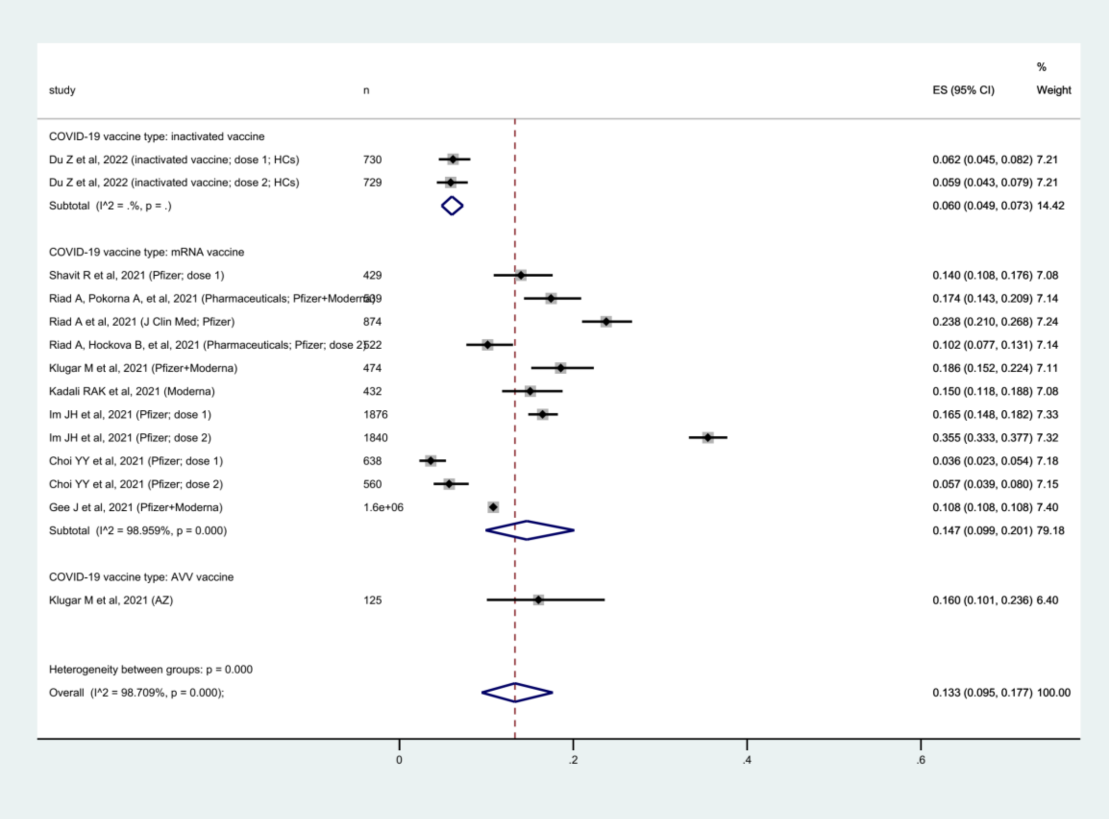

Figure S16. Incidence of local injection site swelling for different types of COVID-19 vaccines

AVV vaccine, adenovirus viral vector vaccine.

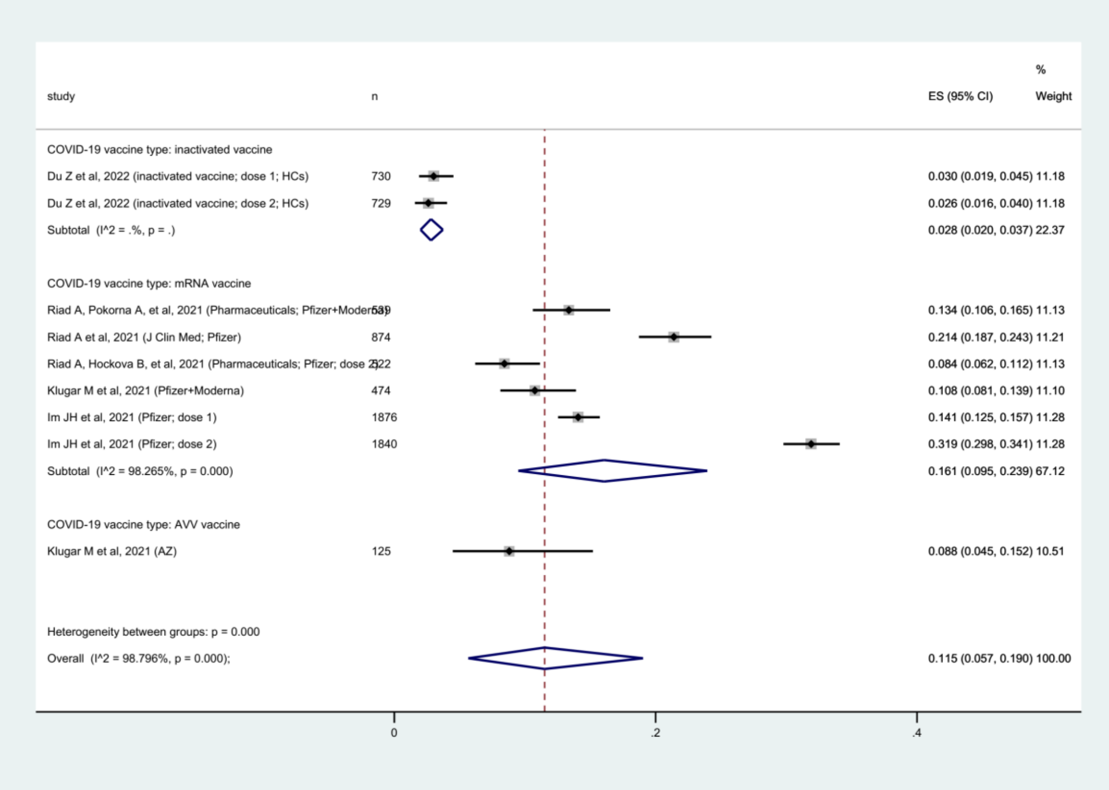

Figure S17. Incidence of local injection site redness for different types of COVID-19 vaccines

AVV vaccine, adenovirus viral vector vaccine.

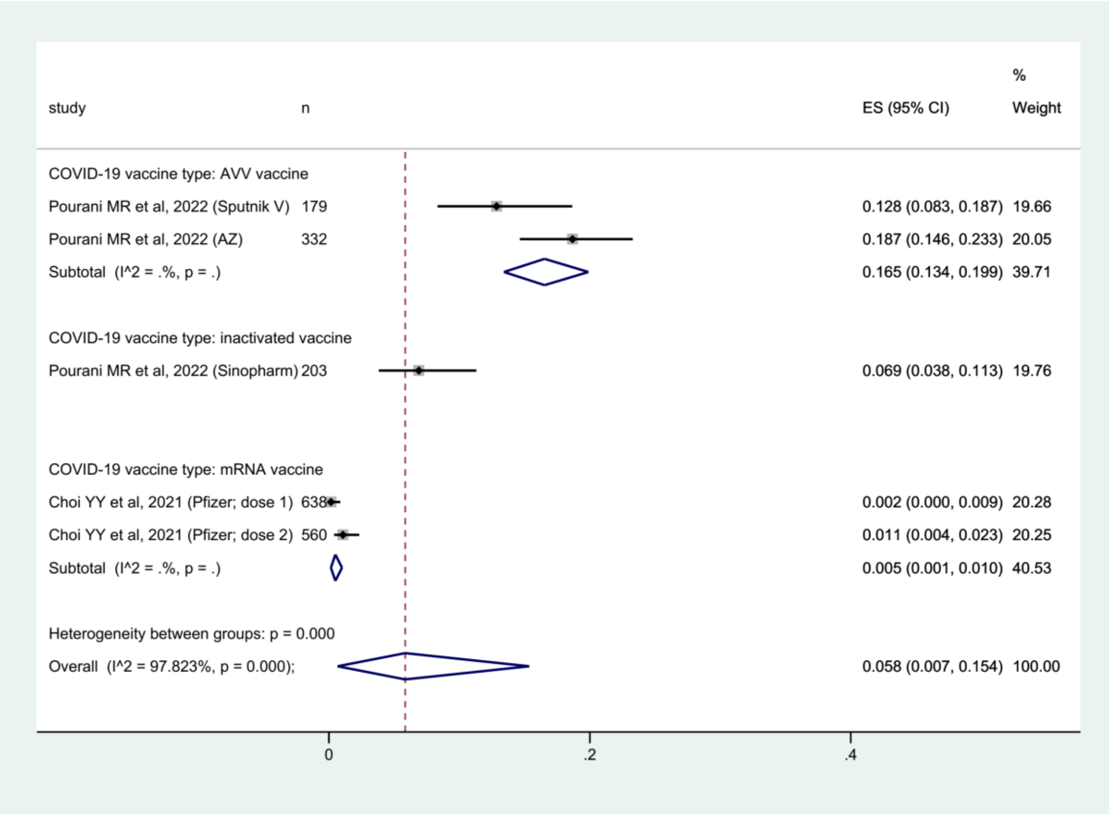

Figure S18. Incidence of local injection site erythema for different types of COVID-19 vaccines

AVV vaccine, adenovirus viral vector vaccine.

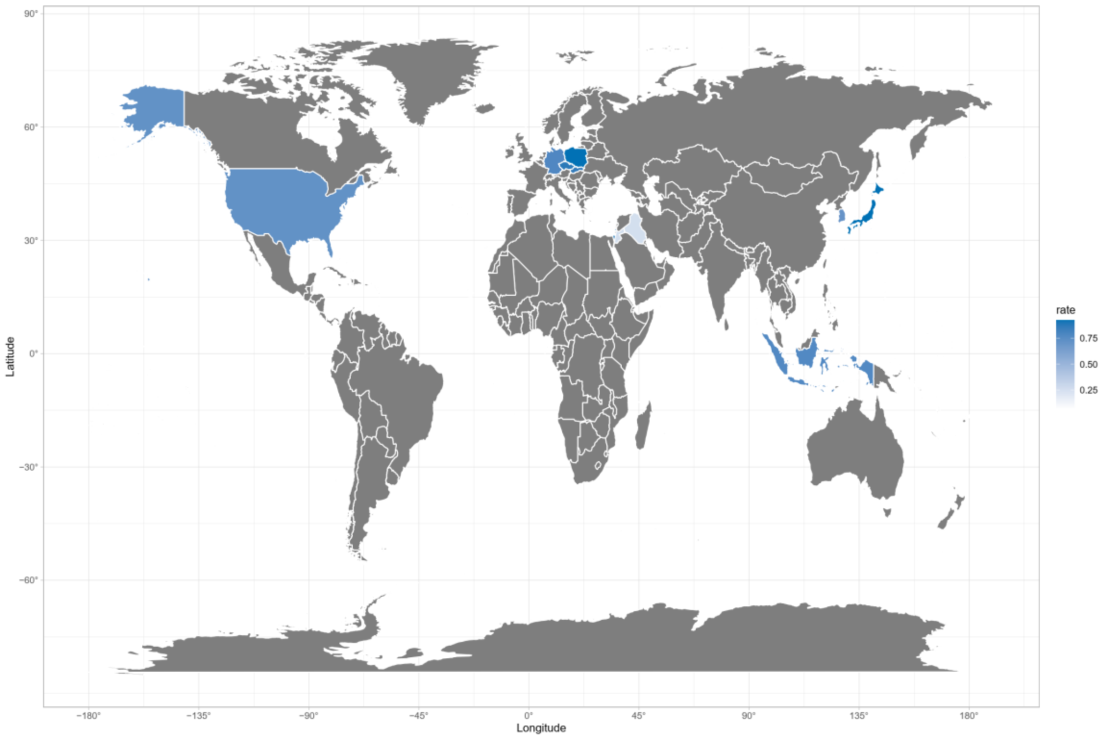

Figure S19. Global map for incidence of overall local injection site reactions related to mRNA vaccines

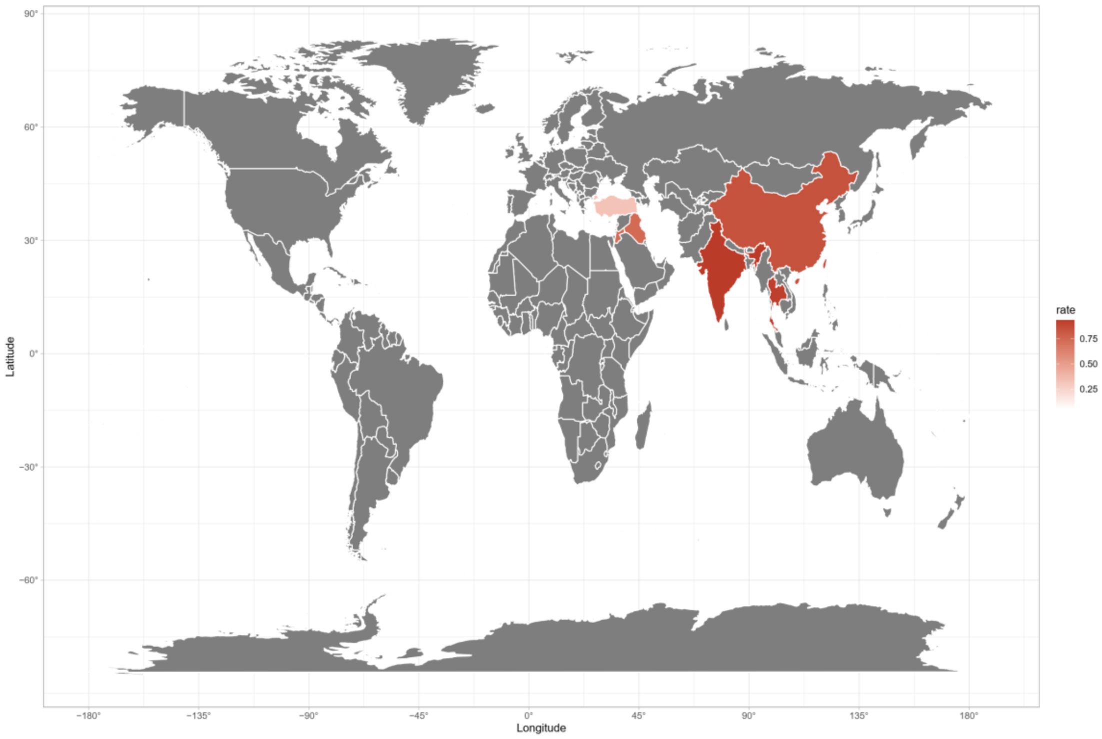

Figure S20. Global map for incidence of overall local injection site reactions related to inactivated virus vaccines

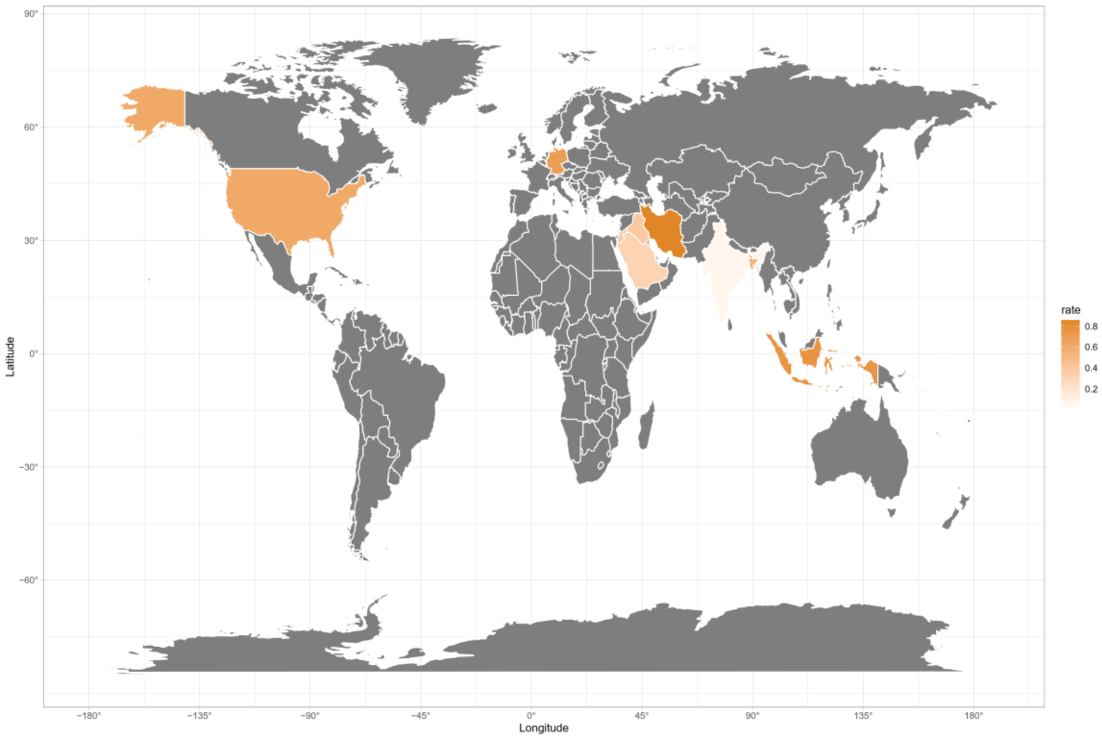

Figure S21. Global map for incidence of overall local injection site reactions related to adenovirus viral vector vaccines

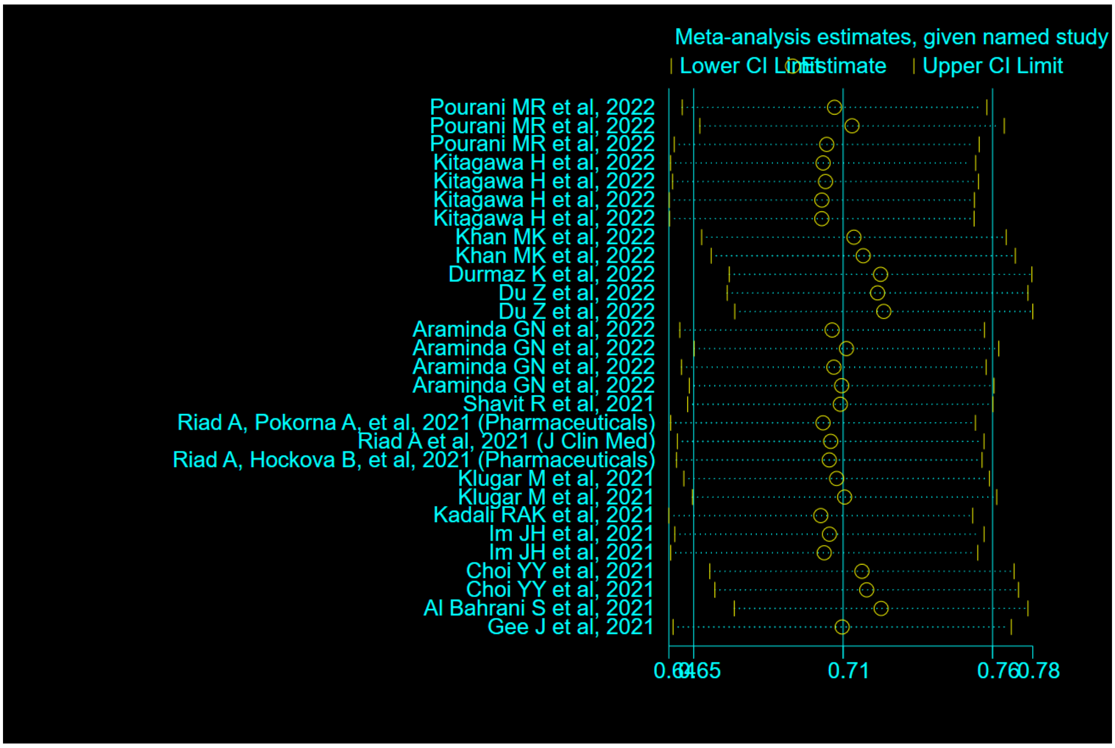

Figure S22. Sensitivity analysis for overall local injection site reactions

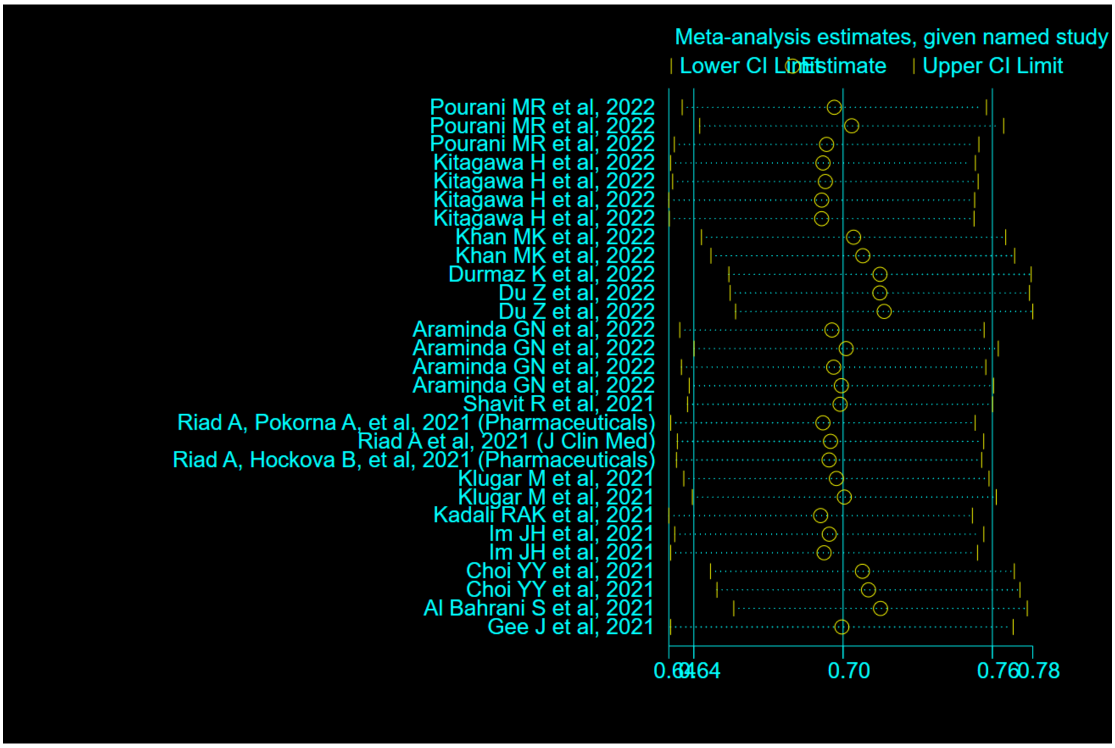

Figure S23. Sensitivity analysis for local injection site pain

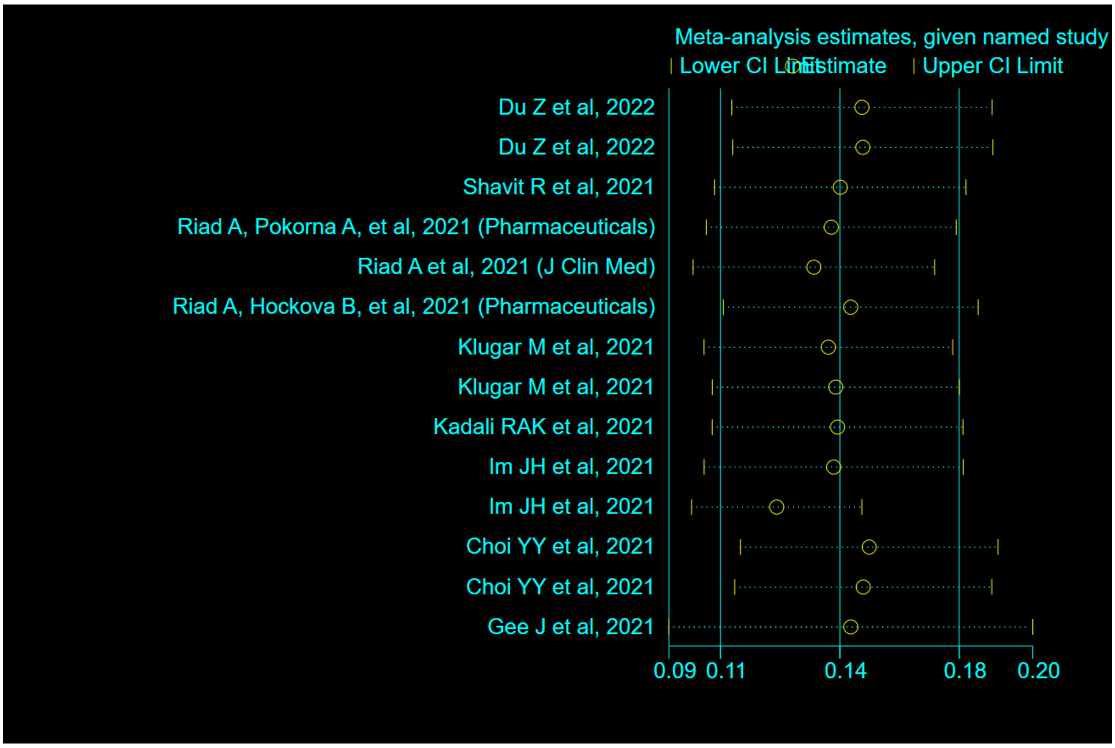

Figure S24. Sensitivity analysis for local injection site swelling

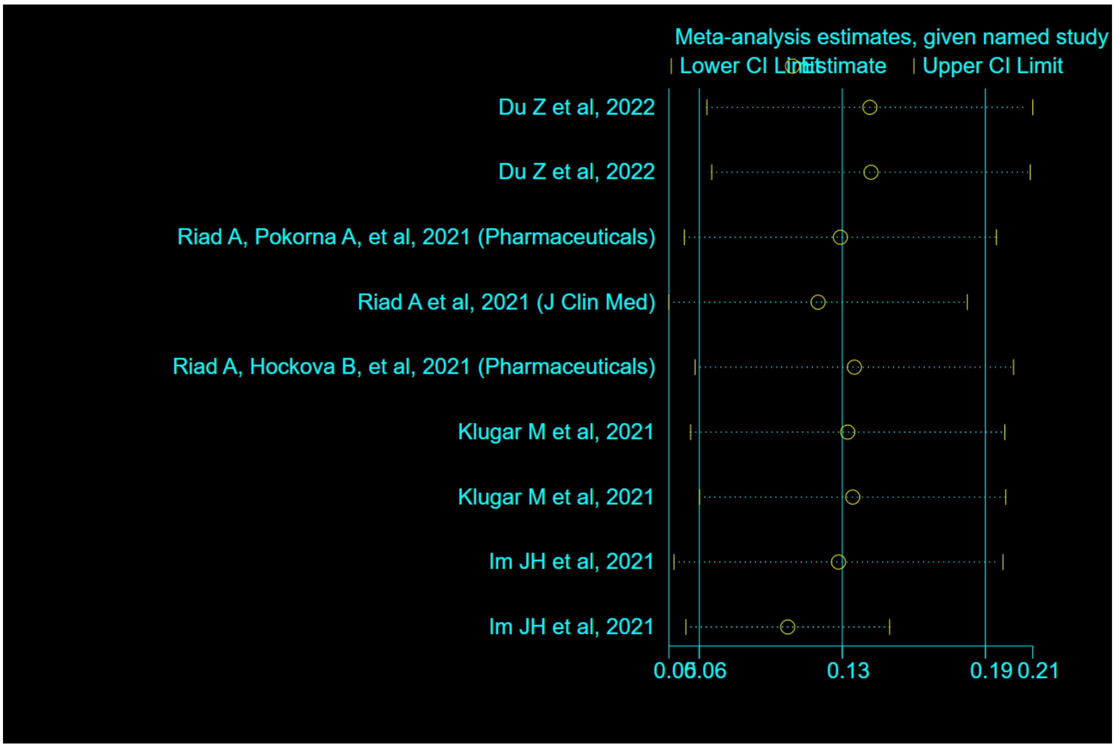

Figure S25. Sensitivity analysis for local injection site redness

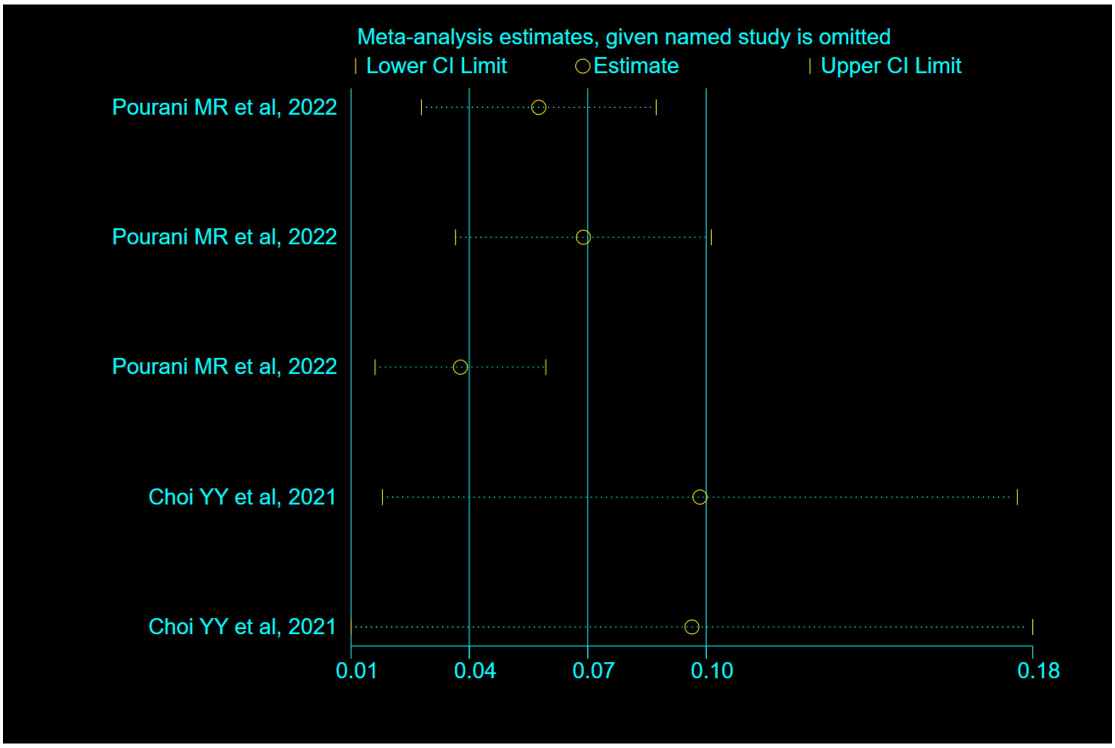

Figure S26. Sensitivity analysis for local injection site erythema

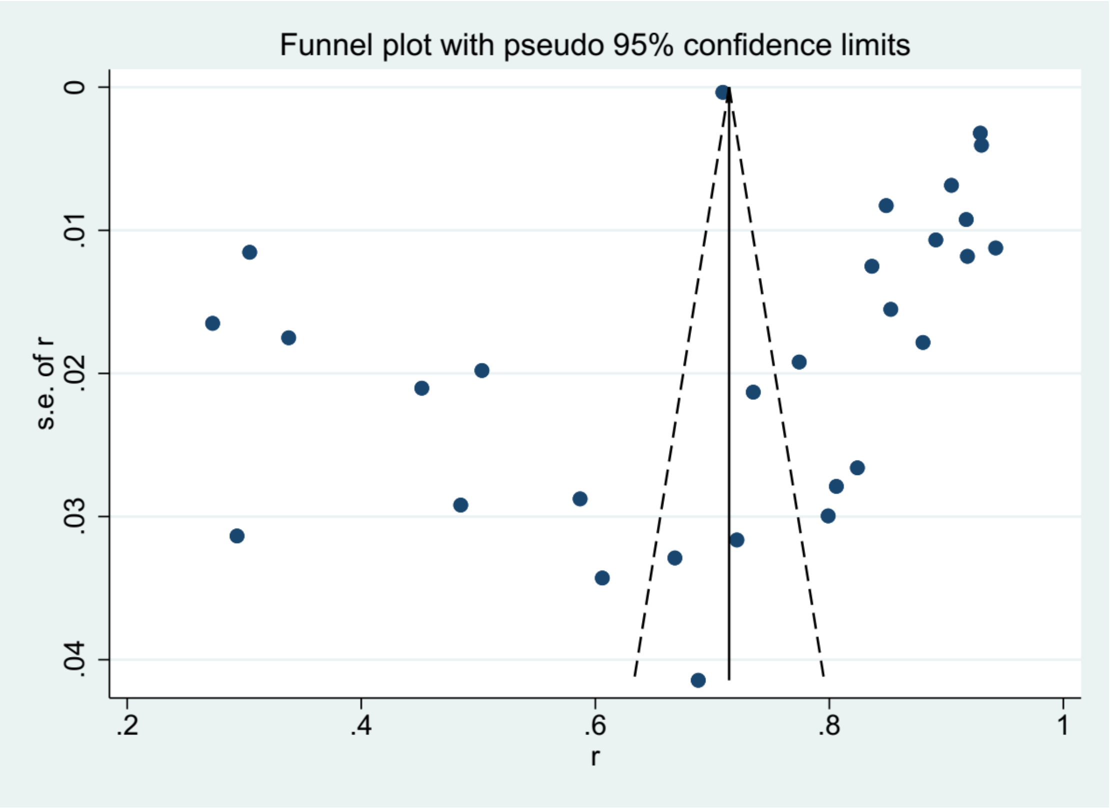

Figure S27. Funnel plots for overall local injection site reactions

P value for Egger's test = 0.631 and *P* value for Begg's test <0.001

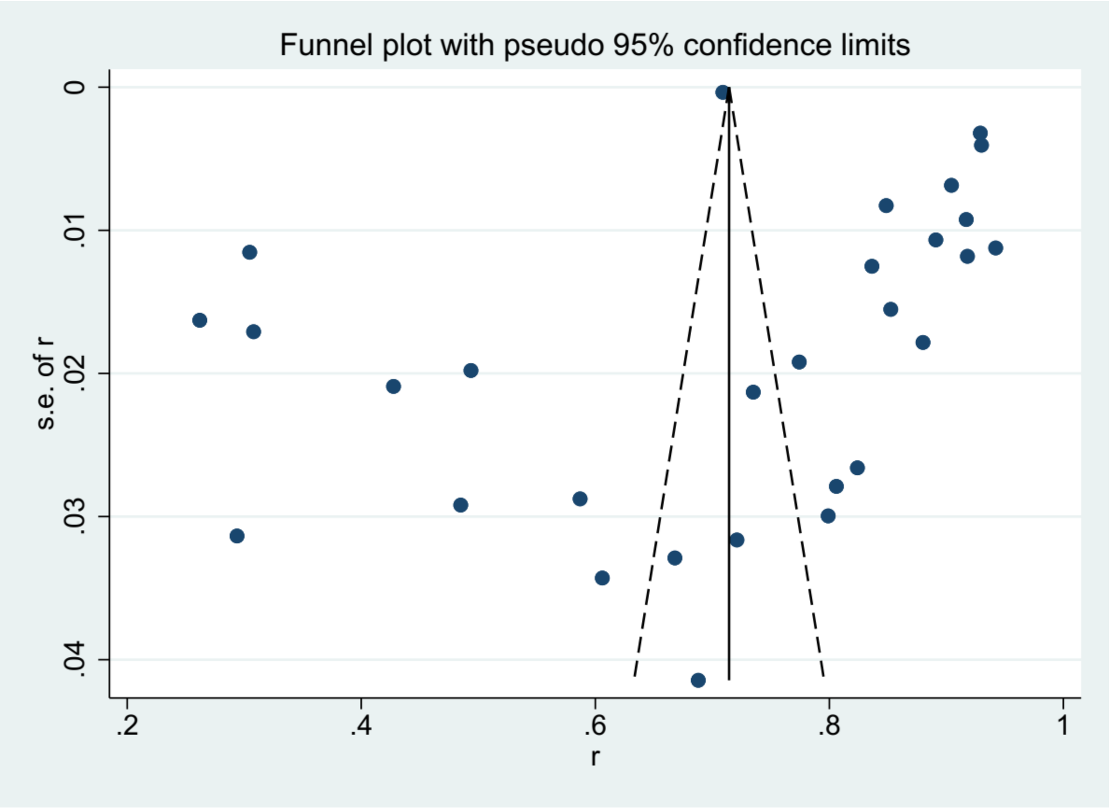

Figure S28. Funnel plots for local injection site pain

P value for Egger's test = 0.669 and *P* value for Begg's test <0.001

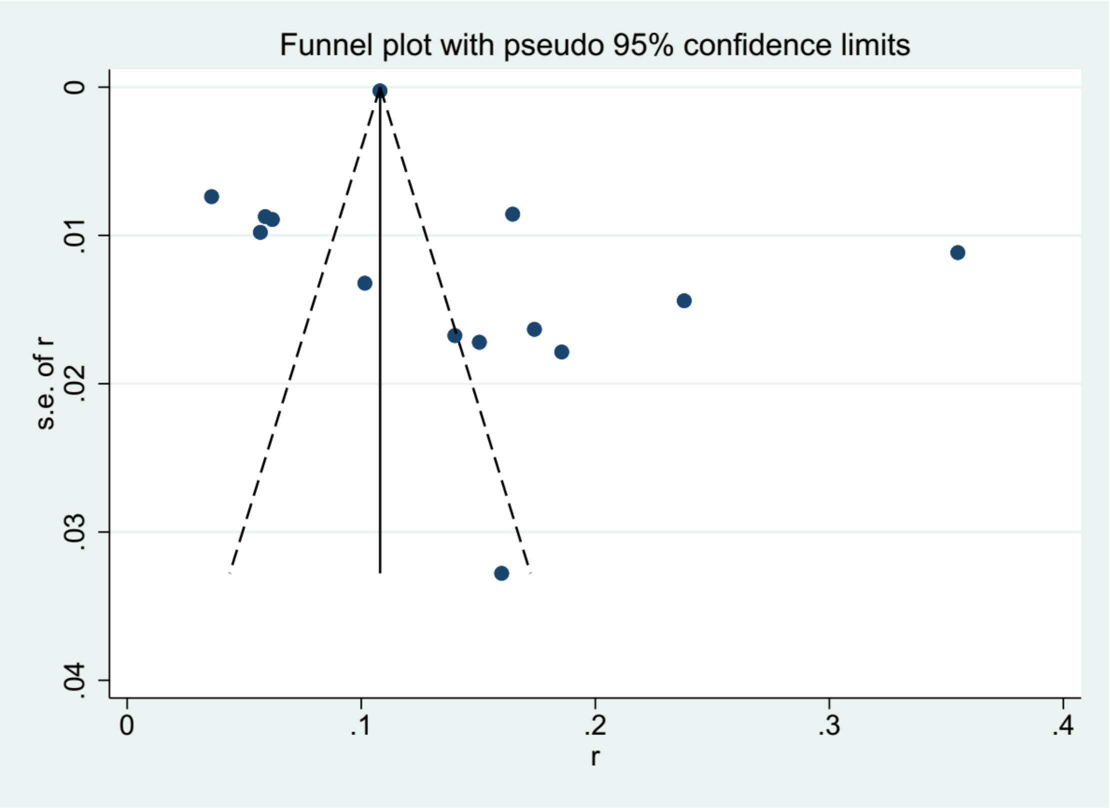

Figure S29. Funnel plots for local injection site swelling

P value for Egger's test = 0.201 and *P* value for Begg's test =0.010

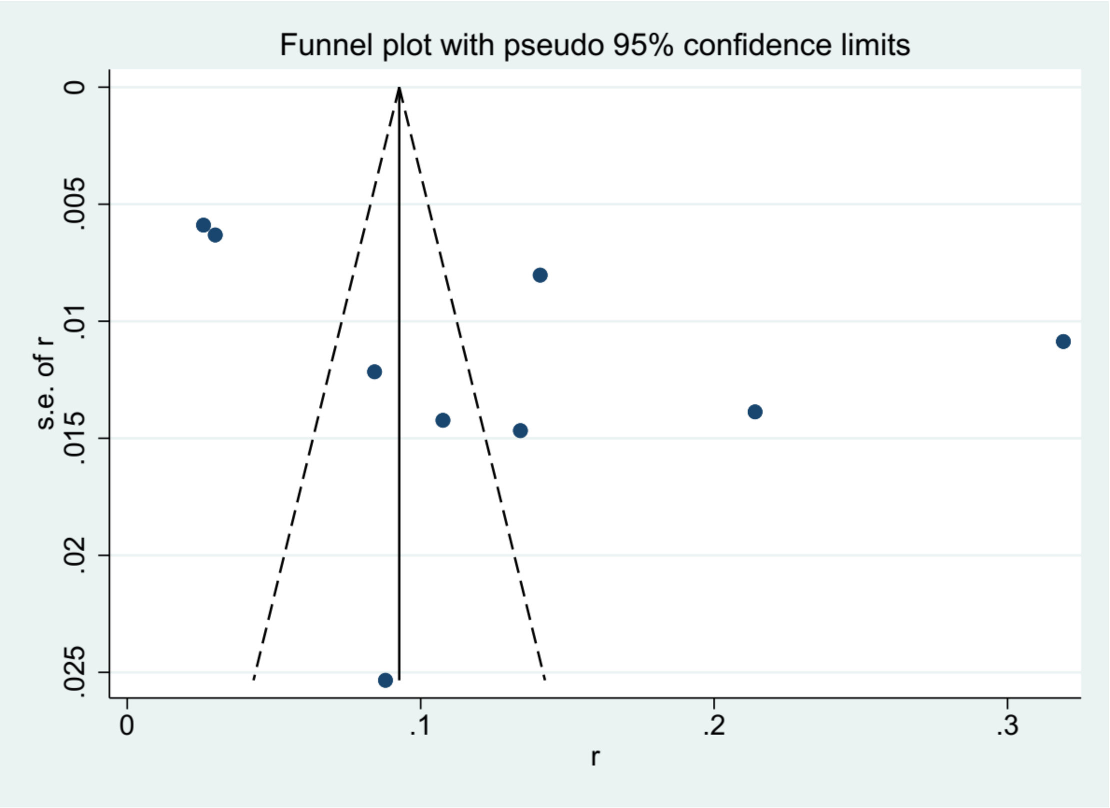

Figure S30. Funnel plots for local injection site redness

P value for Egger's test = 0.003 and *P* value for Begg's test = 0.144

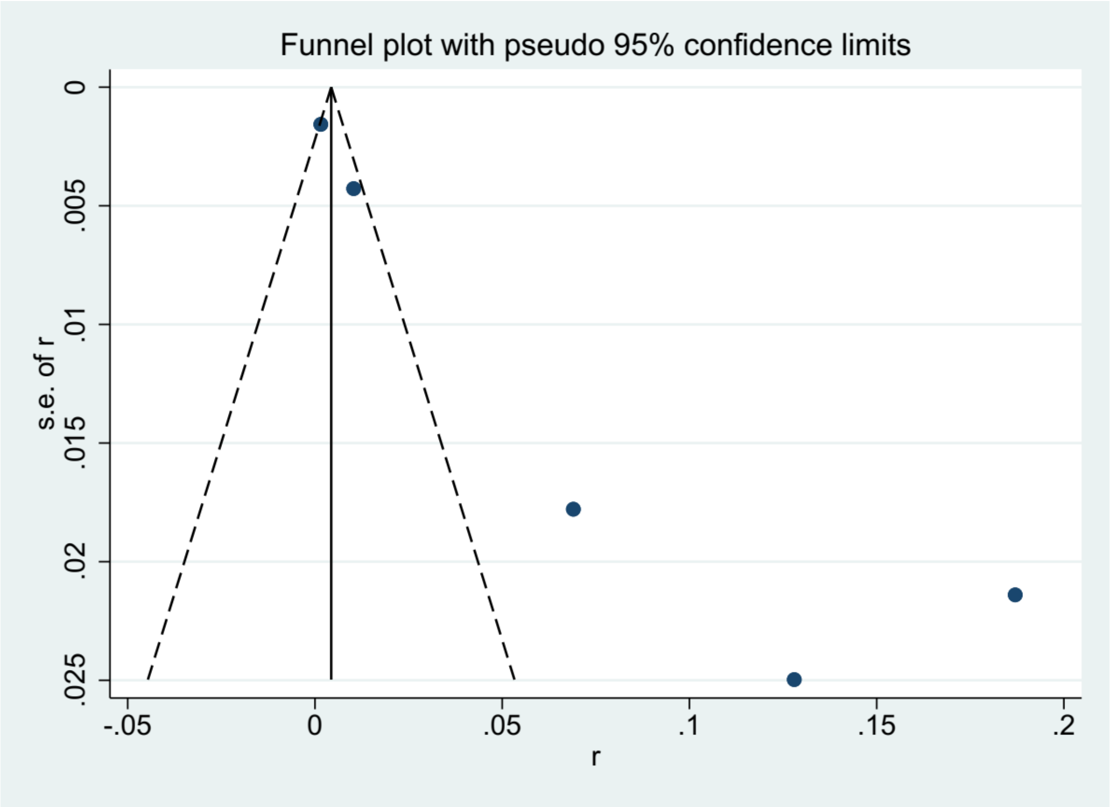

Figure S31. Funnel plots for local injection site erythema

P value for Egger's test = 0.015 and *P* value for Begg's test = 0.050
